# A comparative analysis of methylome profiles of *Campylobacter jejuni* sheep abortion isolate and gastroenteric strains using PacBio data

**DOI:** 10.3389/fmicb.2014.00782

**Published:** 2015-01-14

**Authors:** Kathy T. Mou, Usha K. Muppirala, Andrew J. Severin, Tyson A. Clark, Matthew Boitano, Paul J. Plummer

**Affiliations:** ^1^Department of Veterinary Microbiology and Preventive Medicine, College of Veterinary Medicine, Iowa State UniversityAmes, IA, USA; ^2^Genome Informatics Facility, Office of Biotechnology, Iowa State UniversityAmes, IA, USA; ^3^Pacific BiosciencesMenlo Park, CA, USA; ^4^Department of Veterinary Diagnostic and Production Animal Medicine, College of Veterinary Medicine, Iowa State UniversityAmes, IA, USA

**Keywords:** methylation, *Campylobacter jejuni*, LuxS, quorum sensing, methylome

## Abstract

*Campylobacter jejuni* is a leading cause of human gastrointestinal disease and small ruminant abortions in the United States. The recent emergence of a highly virulent, tetracycline-resistant *C. jejuni* subsp. *jejuni* sheep abortion clone (clone SA) in the United States, and that strain's association with human disease, has resulted in a heightened awareness of the zoonotic potential of this organism. Pacific Biosciences' Single Molecule, Real-Time sequencing technology was used to explore the variation in the genome-wide methylation patterns of the abortifacient clone SA (IA3902) and phenotypically distinct gastrointestinal-specific *C. jejuni* strains (NCTC 11168 and 81-176). Several notable differences were discovered that distinguished the methylome of IA3902 from that of 11168 and 81-176: identification of motifs novel to IA3902, genome-specific hypo- and hypermethylated regions, strain level variability in genes methylated, and differences in the types of methylation motifs present in each strain. These observations suggest a possible role of methylation in the contrasting disease presentations of these three *C. jejuni* strains. In addition, the methylation profiles between IA3902 and a *luxS* mutant were explored to determine if variations in methylation patterns could be identified that might explain the role of LuxS-dependent methyl recycling in IA3902 abortifacient potential.

## Introduction

As a major cause of human gastroenteritis worldwide, *Campylobacter jejuni* is responsible for over 400 million cases of diarrhea each year (Ruiz-Palacios, 2007) and is among the leading causes of foodborne disease related hospitalizations in the United States (Scallan et al., 2011). In the past two decades, a highly virulent *C. jejuni* clone, named clone SA, has emerged to become the predominant cause of *Campylobacter*-associated sheep abortions in the United States (Sahin et al., 2008). This clone is tetracycline-resistant, leaving drug treatment options limited as tetracycline is the only approved class of antibiotics for treating sheep abortions (Sahin et al., 2008). In addition, recent findings suggest a zoonotic potential for transmission of clone SA from animals to humans (Sahin et al., 2012). Though there is only evidence linking clone SA with human gastroenteritis cases, its ability to cause more severe disease in humans cannot be ruled out.

Concurrently, the widespread emergence of antibiotic resistant *Campylobacter* in both human and animal medicine, combined with limited alternative treatment options has shifted research efforts toward identifying alternative therapeutic and preventative strategies against *Campylobacter* (Luangtongkum et al., 2009). This underscores the importance of improving our understanding of *Campylobacter* pathogenesis to develop appropriate and effective novel treatment interventions.

A recent study by Wu et al. (2013) found that the genome of IA3902, a clinical isolate of clone SA, is remarkably syntenic with that of *C. jejuni* subsp. *jejuni* gastroenteric strains NCTC 11168 (Parkhill et al., 2000) and to a lesser extent that of 81-176 (Russell et al., 1989). The pVir plasmids of IA3902 and 81-176 are also syntenic (Wu et al., 2013). However, the disease presentations between the gastroenteric strains 11168 and 81-176, and abortigenic IA3902 are very different. More specifically, our research group demonstrated that 11168 would not induce abortion following oral inoculation in the pregnant guinea pig model (Burrough et al., 2009). The differences in disease presentation were found not due to the presence of major pathogenicity islands or virulence genes associated with an abortion phenotype (Wu et al., 2013). However, comparative genomic analysis by Wu et al. (2013) identified several differences in global gene expression profiles between IA3902 and 11168, which were attributed to small genomic changes within the chromosomes, including a large number of single-nucleotide polymorphisms and indels. We expanded on this hypothesis and propose that DNA methylation may explain the differences in disease presentation in *C. jejuni*.

Enzymes that carry out DNA methylation activities are part of restriction-modification (R-M) systems, which are best known for their role in prokaryotic defense mechanisms (Vasu and Nagaraja, 2013). R-M systems are grouped into four classes (Types I–IV) and are classified based on the enzyme composition and associated cofactors, specific base position methylated in the recognized sequence motif, and the symmetry of the motif on the double-stranded DNA (Roberts et al., 2003). In addition to prokaryotic defenses, methylation serves numerous other important roles associated with gene expression and regulation, cell maintenance, and virulence (Marinus and Casadesus, 2009).

We first explored whether the methylation patterns between IA3902 and *C. jejuni* strains 11168 (Parkhill et al., 2000) and 81-176 (Russell et al., 1989) were different. This was determined by using Pacific Biosciences' Single-Molecule, Real-Time (SMRT) sequencing technology (Flusberg et al., 2010) to characterize the genome methylation patterns for IA3902. Previous methods for detecting DNA methylation and other common epigenetic markers at the genomic level have been difficult due to the lack of quick and simple methods sensitive enough to detect such markers (Korlach and Turner, 2012). However, the advent of Pacific Biosciences' SMRT sequencing has made it possible to detect such markers quickly and directly map genome-wide methylation patterns of bacteria (Davis et al., 2013). We utilized the methylation data of 11168 and 81-176 from a recent publication (Murray et al., 2012) to compare the methylation profiles with IA3902.

Previously, we reported that a *luxS* mutation in IA3902 significantly lowers its virulence (Plummer et al., 2012). Therefore, the mutation in the LuxS enzyme and its effect on the methylation of IA3902 was also investigated. *C. jejuni* possess the Autoinducer-2/LuxS system, which is well-known in other bacterial species for its quorum sensing role as well as a promising drug target candidate (Sintim et al., 2010). A study from our group found the *luxS* mutation compromised the abortion phenotype of IA3902, when administered orally in a pregnant guinea pig model, but not intraperitoneally (Plummer et al., 2012). The fact that the *luxS* mutant was unable to cause abortions when inoculated orally in the guinea pig model suggests that the mutant was compromised in its ability to colonize, invade the enteric epithelium, and enter systemic circulation (Plummer et al., 2012).

Interestingly, LuxS is a dual-purpose enzyme that is also an important component of the activated methyl cycle (AMC), which is a primary source of methyl groups for DNA methylation (Parveen and Cornell, 2011). In *C. jejuni*, studies have already shown the importance of methylation for virulence expression, such as adhesion and invasion of host intestinal epithelia (Kim et al., 2008; Anjum, 2013). These two traits coincide with those hypothesized to be affected by the *luxS* mutation in the pregnant guinea pig sheep abortion model (Plummer et al., 2012). Based on these observations, we hypothesize that the *luxS* mutation could impact methylation and ultimately attenuate the virulence expression and abortion phenotype of IA3902.

The work in this study provides a comprehensive analysis of the methylome of IA3902. In addition, the in-depth analysis of the methylation motif distributions and genes located within these hyper- and hypomethylated regions of interest together provide extensive insights into the pathobiology of these *C. jejuni* strains.

## Materials and methods

### Bacterial strains and growth conditions

*C. jejuni* subsp. *jejuni* IA3902 is a clinical isolate of clone SA and its full genome sequence has been determined (Wu et al., 2013). IA3902 and its LuxS mutant (IA3902Δ*luxS*) (Plummer et al., 2012) were routinely grown in Mueller–Hinton broth or agar incubated in gas jars at 42°C in a microaerobic environment (5% O_2_, 10% CO_2_, 85% N_2_) until 24 h (stationary growth phase). *C*. *jejuni* subsp. *jejuni* NCTC 11168 and 81-176 both originated from human gastroenteritis, have been genome-sequenced, and are commonly studied by researchers around the world (Parkhill et al., 2000; Hofreuter et al., 2006).

### Genomic DNA isolation and preparation for sequencing

DNA extraction from IA3902 and IA3902Δ*luxS* was performed using Wizard Genomic DNA purification kit (Promega, Madison, WI). The NanoDrop ND-1000 spectrophotometer (Thermo Scientific, Wilmington, DE) and Qubit fluorometer (Life Technologies, Grand Island, NY) were used to measure DNA quantity and quality. Approximately 10 ug of genomic DNA per strain were sent to Pacific Biosciences for library preparation and SMRT sequencing.

### Sequencing library preparation and SMRT sequencing

Preparation of IA3902 and IA3902Δ*luxS* DNA samples for Single-Molecule, Real-Time (SMRT) sequencing was performed as previously described (Travers et al., 2010; Clark et al., 2012). Genomic DNA was randomly sheared to approximately 15 kb using gTUBEs (Covaris, Inc., Woburn, MA). Fragmented DNA was damage repaired, end repaired, and ligated to hairpin adapters using standard SMRTbell template preparation protocols (Pacific Biosciences, Menlo Park, CA). SMRTbell templates with sizes greater than 5–10 kb were size-selected with Blue Pippin (Sage Science, Beverly, MA). SMRT sequencing was carried out using P4/C2 chemistry with 4 SMRT Cells per sample. Genomes were assembled into single contigs using the HGAP algorithm as part of the SMRT analysis suite v2.0 (Chin et al., 2013).

### Bioinformatic analysis of methylation motifs

Identification of methylated motifs from SMRT sequencing data on IA3902, IA3902Δ*luxS*, 11168, and 81-176 was performed using the SMRT analysis suite v2.0 as previously described (Murray et al., 2012). Methylated motif information from other *Campylobacter* strains was obtained from the Restriction Enzyme database (REBASE) (Roberts et al., 2010). The output from SMRT sequencing analysis also included each motif's methylation site, methylation score and the extent of genome methylation for each sequence motif (Supplementary Datasheets 2–5).

Based on the methylation data, and genome annotations available for IA3902 and *C. jejuni* strains in NCBI, genes that were methylated in each strain were identified (Quinlan and Hall, 2010). The methylation motifs associated with each gene were also analyzed in this paper. For every gene, we assigned a functional group or role category as described elsewhere (Wu et al., 2013). Then, for every methylation motif, we obtained the total number of methylated genes in each role category.

### Bioinformatic analysis of whole-genome methylation motif distribution plots

The statistical software package R was used to generate methylation motif distribution plots for the whole genome of each strain using a bin size of 1000 bp. Methylation sites on both strands of the DNA were considered. Mean and standard deviations are reported in the results. In this study, bins that have more than 41–45 methylation sites (the actual number mean + three times standard deviation varies for each strain) per bin were considered as hypermethylated regions. Bins that have 4 or less methylation sites were considered as hypomethylated regions.

## Results

### SMRT sequencing and methylome analysis of IA3902

SMRT sequencing of IA3902 yielded one circular chromosome (1.64 Mb) and one circular plasmid (0.037 Mb). Its genome encoded seven total recognition sites for methylation, also known as sequence motifs (Table 1). At least 98.8% of all seven motifs present in the genomes were detected by SMRT sequencing. We found two pairs of bipartite motifs (TAAYN_5_TGC/GCAN_5_RTTA, and GAGN_5_RTG/CAYN_5_CTC), a palindromic motif (RAATTY/YTTAAR), and two non-paired motifs (CAAAYG and GAAGAA) (methylated adenines are underlined). Motifs were compared to predicted IA3902 data in the Restriction Enzyme Database REBASE (http://rebase.neb.com/rebase/rebase.html), a web-based database containing comprehensive information about all genes, enzymes, and genomes involved in DNA restriction and modification (Roberts et al., 2010). Each bipartite motif is recognized and modified by the same enzyme (e.g., TAAYN_5_TGC is recognized by the R-M enzyme CjeIAORF994P, and GAGN_5_RTG is recognized by M.CjeIAII).

**Table 1:** **Methylome motifs detected within the *C. jejuni* IA3902 genome**.

**Motif^a^**	**Modification type**	**# Of motifs detected**	**# Of motifs in genome**	**% Motifs detected**	**Partner motif**	**R-M enzymes^d^^,^^e^**
GAGNNNNNRTG^b^	m6A	717	717	100	CAYNNNNNCTC	CjeIAORF994P
CAYNNNNNCTC^b^	m6A	716	717	99.86	GAGNNNNNRTG	CjeIAORF994P
CAAAYG	m6A	1760	1760	100		CjeIAORF654P
TAAYNNNNNTGC^c^	m6A	499	499	100	GCANNNNNRTTA	M.CjeIAII
GCANNNNNRTTA^c^	m6A	493	499	98.8	TAAYNNNNNTGC	M.CjeIAII
GAAGAA	m6A	2557	2563	99.77		CjeIAORF32P
RAATTY	m6A	27318	27514	99.29	RAATTY	M.CjeIAI

a *Sequence motifs are listed in the 5 ′ to 3 ′ direction. Underlined bases indicate the methylated base on the sequence. Motifs containing Y have either T or C nucleotide, while motifs with R have an A or G nucleotide*.

b,c *Complementary motif sequences*.

d *Last column lists assigned restriction-modification (R-M) enzymes predicted to recognize respective motif(s)*.

e *The motifs and associated methyltransferases (MTases) are excellent candidates for the respective R-M system types. However, because the designations are not definitive, further tests are required to confirm the functional statuses of the MTases*.

The motifs CAAAYG and GAAGAA were novel discoveries as they have not been previously predicted for IA3902 in REBASE. Both motifs have been submitted to REBASE for inclusion in the database. These were also the only motifs methylated on just one DNA strand while all other motifs were methylated on both strands. The methylome of IA3902 revealed that more than 98% of the identified motifs were modified. More specifically, the adenine base was the only base methylated in the sequence motifs (Table 1). This type of modification, N6-methyladenine (m6A), was also the only form of base methylation found in two commonly studied *C. jejuni* subsp. *jejuni* gastroenteric strains NCTC 11168 and 81-176 in an earlier study (Murray et al., 2012).

Five enzymes were identified that are responsible for the m6A methylation on the seven motifs (Table 2). The enzymes detected in the IA3902 strains are part of Types I or II R-M systems. Type I systems are enzyme complexes made up of subunits with restriction (R), methylation (M), and specificity (S) activities (Vasu and Nagaraja, 2013). In this study, M.CjeIAII was the only known Type I methyltransferase (MTase). On the other hand, Type II systems are simpler and ubiquitous (Bujnicki, 2001). They encode two proteins with separate activities: one as an endonuclease and the other as a MTase. In this case, all other MTases identified in this study were Type II, including CjeIAORF32P, M.CjeIAI, CjeIAORF654P, and CjeIAOF994P.

**Table 2:** **Putative Restriction-Modification (RM) systems detected in IA3902**.

**R-M system type**	**Locus**	**Gene^a^**	**Name^a^^,^^b^**	**Gene description**	**Associated sequence motif (5′ to 3′ direction)**	**Partner motif**	**Sequence motif identified in this study?**
I	CJSA_1465	R	CjeIAIIP	Putative restriction type I enzyme R protein	TAAYNNNNNTGC	GCANNNNNRTTA	Yes
I	CJSA_1467	S	CjeIAII	Putative restriction type I enzyme S protein	TAAYNNNNNTGC	GCANNNNNRTTA	Yes
I	CJSA_1469	M	CjeIAII	Putative restriction type I enzyme M protein	TAAYNNNNNTGC	GCANNNNNRTTA	Yes
II	CJSA_0032	RM	CjeIAORF32P	Type II restriction modification enzyme	Unknown (putatively GAAGAA)		Yes
II	CJSA_0199	M	CjeIAI	D12 N6 adenine-specific DNA methyltransferase	RAATTY	RAATTY	Yes
II	CJSA_0654	RM	CjeIAORF654P	Putative restriction modification enzyme	Unknown (putatively CAAAYG)		Yes
II	CJSA_0994	RM	CjeIAORF994P	Restriction modification enzyme	GAGNNNNNRTG	CAYNNNNNCTC	Yes

a *The Gene and Names of the systems are given as assigned by REBASE following submission of the data*.

b *The motifs and associated MTases are excellent candidates for the respective R-M system types. However, because the designations are not definitive, further tests are required to confirm the functional statuses of the MTases*.

As of recently, three of the detected motifs (TAAYN_5_TGC, RAATTY, and GAGN_5_RTG) have already been reported in REBASE. These three motifs were also predicted to be recognized by three IA3902 R-M enzymes (M.CjeIAII, M.CjeIAI, and CjeIAOF994P, respectively). There was, however, no mention of any IA3902 R-M enzyme that recognized GAAGAA and CAAAYG. We predicted that the R-M enzymes CjeIAORF32P and CjeIAORF654P would recognize GAAGAA and CAAAYG, respectively (Table 2). This prediction was based on CjeIAORF654P having 97% and 96% DNA sequence identity with previously characterized R-M enzymes of other *Campylobacter jejuni* strains, CjeFIII (*C. jejuni* 81-176) and CjeNIII (*C. jejuni* NCTC 11168) (Supplementary Datasheet 1, Table 3). Since the closest IA3902 motif to these two motifs was CAAAYG, we thus predicted that CjeIAORF654P would recognize CAAAYG. As the remaining R-M enzyme without a candidate motif assigned to it, CjeIAORF32P was predicted to recognize GAAGAA by default (Supplementary Datasheet 1, Table 5). However, these were only predictions and the function of the MTases will need to be tested for confirmation. Thus far, the results have revealed novel information about the forms of methylation and associated motifs found in IA3902.

### Comparative analysis of IA3902 motifs with *C. jejuni* gastroenteric strains 11168 and 81-176

Evidence for differing methylation patterns between closely-related *C. jejuni* strains NCTC 11168 and 81-176 (Murray et al., 2012) prompted our investigation for comparisons with IA3902. The results from Wu et al. (2013) suggested that small genomic changes are cause for differences in global gene expression profiles and thus disease presentations. One such small genomic change is DNA methylation, which lead to the hypothesis that IA3902 methylome profile will be different from the gastroenteric strains and possibly explain for the unique hyper virulence of each strain. Supplementary Datasheet 1, Tables 1–5 show a list of all motifs known and detected in this study for *C. jejuni* strains IA3902, 11168, and 81-176. In addition, the associated MTases of the motifs are listed.

Of the motifs identified, the following were identical or homologous between the 3 strains (Supplementary Datasheet 1, Tables 1–3): TAAYN_5_TGC/GCAN_5_RTTA, RAATTY, and the set CAAAYG (IA3902), GKAAYG (11168), and GCAAGG (81-176). The motifs GAGN_5_RTG/CAYN_5_CTC (IA3902) and GAGN_5_GT/ACN_5_CTC (11168) were homologous, but only between IA3902 and 11168 (Supplementary Datasheet 1, Table 4). Motifs homologous between only IA3902 and 81-176 included GAAGAA (IA3902) and GGRCA (81-176) (Supplementary Datasheet 1, Table 5). The only motifs that did not have a homolog with IA3902 were GAGAN_4_GMT motif in 11168, and CAAYN_6_ACT/AGTN_6_RTTG motif in 81-176.

When we analyzed each strain's MTases and their associated motifs, we discovered that M.CjeIAII (IA3902) showed 100% identity with M.CjeNIV (11168) and M.CjeFIV (81-176), were all Type I restriction enzymes, and recognized the motif TAAYN_5_TGC (Supplementary Datasheet 1, Table 1). In Supplementary Datasheet 1, Table 2, M.CjeIAI (IA3902) showed 100% identity with M.CjeNI (11168) and M.CjeFI (81-176), and all three enzymes were Type II DNA MTases that recognized the RAATTY motif. CjeIAORF654P (IA3902), as described earlier, showed 96% homology with CjeNIII (11168), and 97% homology with CjeFIII (81-176) (Supplementary Datasheet 1, Table 3). The motifs recognized by these Type II enzymes, GKAAYG (11168) and GCAAGG (CjeFIII of 81-176), were homologous with IA3902 motif CAAAYG.

In Supplementary Datasheet 1, Table 4, the Type II enzyme CjeIAORF994P (IA3902) and CjeNII (11168) were 86% homologous, and both recognized similar motifs: GAGN_5_RTG (IA3902) and GAGN_5_GT (11168). Lastly, CjeIAORF32P (IA3902) had 65% homology with CjeFV (81-176). Since CjeFV recognized the motif GGRCA, we thus grouped it with CjeIAORF32P and its recognized motif GAAGAA (Supplementary Datasheet 1, Table 5). Altogether, each of the seven motifs of IA3902 was homologous with at least one other motif in 11168 and/or 81-176.

### Comparative analysis of methylome distributions of IA3902, *C. jejuni* 11168 and 81-176

The methylomes of IA3902, 11168 and 81-176 contained a total number of methylated bases ranging from 13,835 (81-176), 14,632 (11168), and 15,748 (IA3902). The *ori*C regions of all three strains were realigned to the exact same region (*dnaA* gene from nucleotide positions 1–1323, and the origin of replication, or *ori*C, from nucleotide positions 1324–1482). Although the motifs and MTases were similar between the three strains, distinguishing aspects of each strain's methylome density plot revealed otherwise.

**Figure 1:**
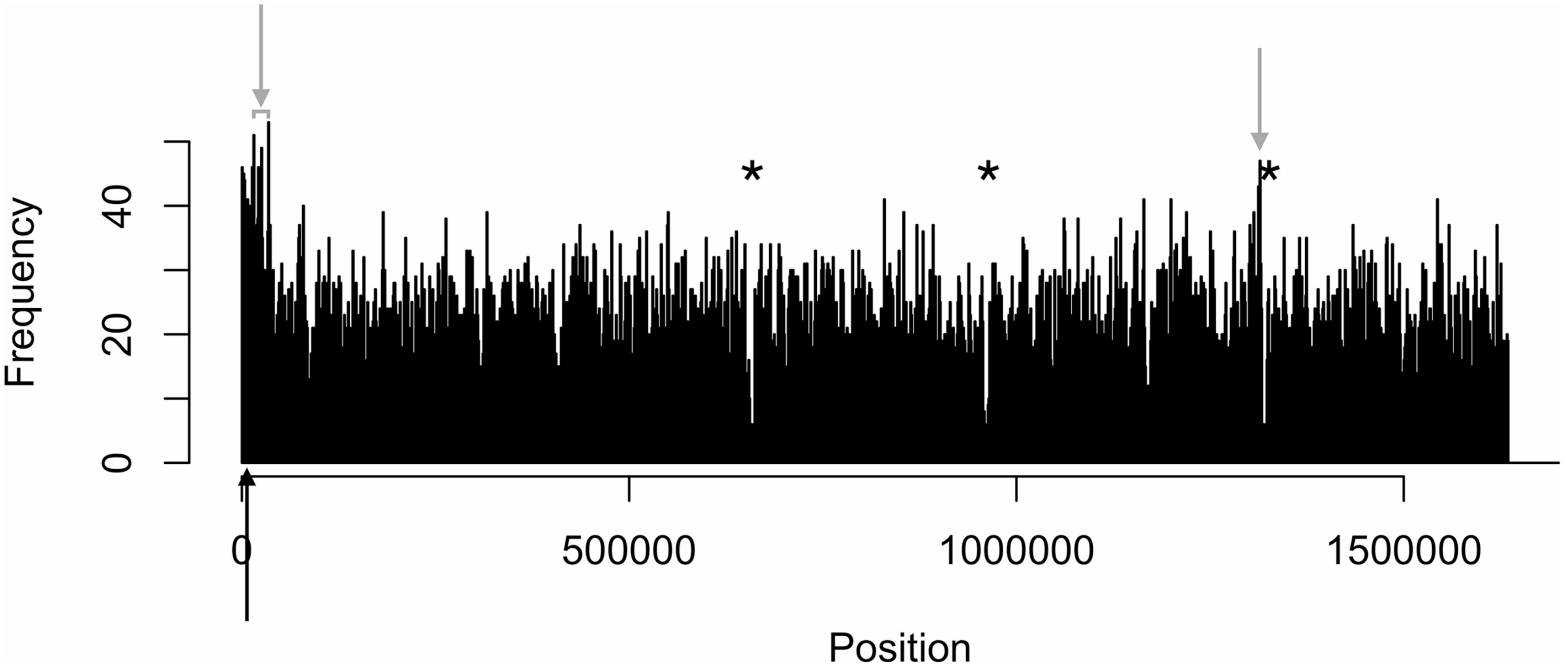
**Distinct areas of hyper- and hypomethylation in the whole-genome methylome plot of IA3902**. Distribution plots of all motifs are combined per 1 kb across the genome. Gray arrows indicate hypermethylated areas, which have methylation frequencies of at least 45 and higher. Asterisks indicate major hypomethylated regions, which contain several adjacent bins with methylation frequencies of 4 or less. Black arrows indicate origin of replication (*oriC*) sites.

**Figure 2:**
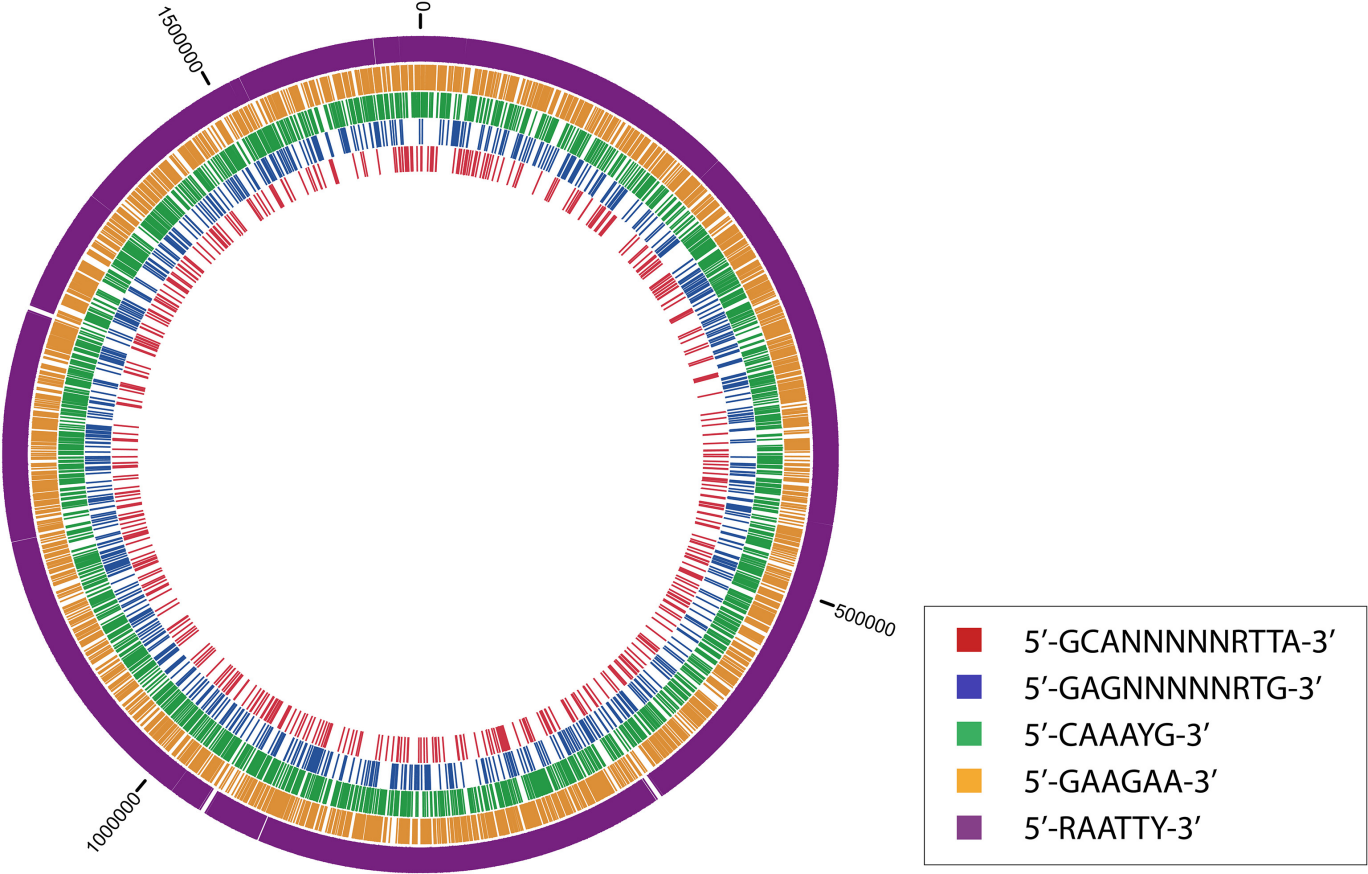
**Circos plot displaying the distributions of each motif across the genome of IA3902**.

**Figure 3:**
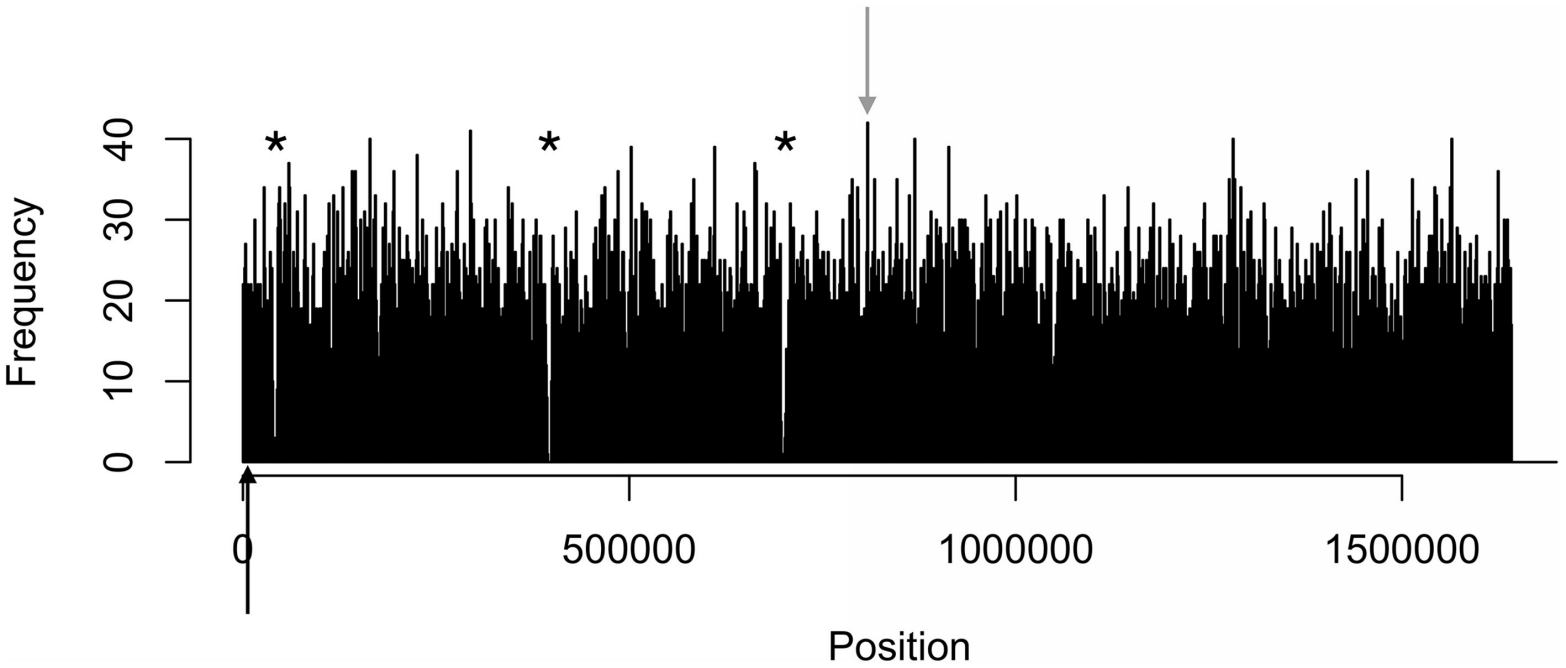
**Distinct areas of hyper- and hypomethylation in the whole-genome methylome plot of 11168**. Distribution plots of all motifs are combined per 1 kb across the genome. Gray arrows indicate hypermethylated areas, which have methylation frequencies of at least 41 and higher. Asterisks indicate major hypomethylated regions, which contain several adjacent bins with methylation frequencies of 4 or less. Black arrows indicate origin of replication (*oriC*) sites.

**Figure 4:**
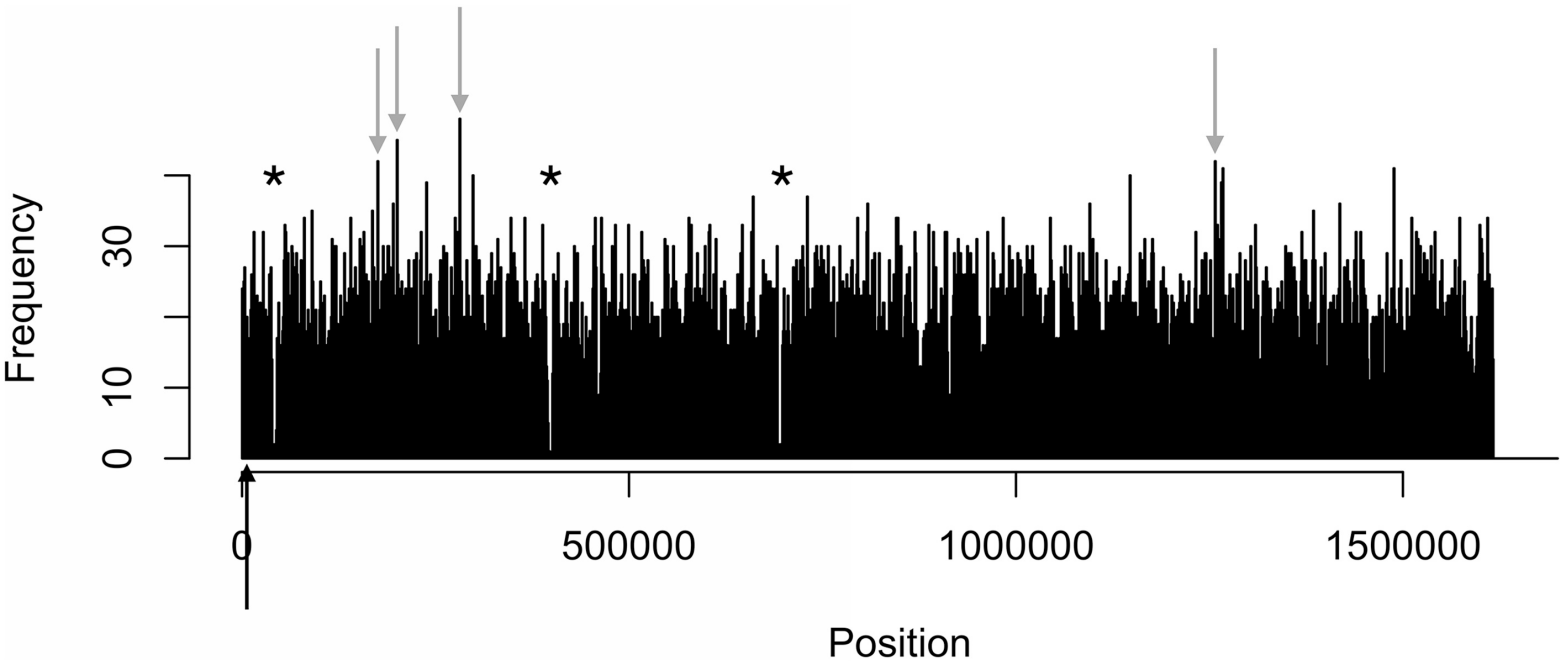
**Distinct areas of hyper- and hypomethylation in the whole-genome methylome plot of 81-176**. Distribution plots of all motifs are combined per 1 kb across the genome. Gray arrows indicate hypermethylated areas, which have methylation frequencies of at least 41 and higher. Asterisks indicate major hypomethylated regions, which contain several adjacent bins with methylation frequencies of 4 or less. Black arrows indicate origin of replication (*oriC*) sites.

The density distributions of all motifs were evaluated across the genomes of each of the three *C. jejuni* strains, shown in Figures 1–**4**. The density distributions of each motif across the genome of IA3902 is shown in Figure 2. The plots showed a general even distribution of all methylation motifs across the genome except for several distinct regions of hyper- and hypo-frequency of methylation motifs. For ease of description, these regions are called hyper- and hypomethylated regions. Genes in a bin with methylation motif frequency values greater than the mean frequency plus three times the standard deviation were deemed hypermethylated genes. The mean frequency values for IA3902, 11168, and 81-176 were 20.15, 18.62, and 18.12, respectively. The standard deviation values were 8.39, 7.55, and 7.92, respectively. Thus, genes would be considered hypermethylated if the number of methylation motifs within these genes (motif frequencies) were greater than 45 (IA3902) or 41 (11168 and 81-176). Genes in a bin with methylation motif frequency values of four or less were considered hypomethylated genes.

### Major hypomethylated regions

Observation of the plots in Figures 1, 3, 4 revealed the presence of three defined hypomethylated regions within the genome of each strain. These regions were considered major hypomethylated areas since they contained multiple adjacent bins that were hypomethylated. The locations of these major hypomethylated areas were clearly different between IA3902 and its gastroenteric counterparts 11168 and 81-176, which are indicated in bold in Supplementary Datasheet 1, Table 6. In addition, the genes at these hypomethylated areas in 11168 and 81-176 were extraordinarily similar while IA3902 had a very different and much wider set of genes (Supplementary Datasheet 1, Table 7).

More specifically, 11168 and 81-176 both had 16S and 23S ribosomal RNA genes in these major hypomethylated areas as shown in Supplementary Datasheet 1, Table 7. 81-176 also had genes coding for hypothetical protein and putative outer-membrane proteins. Genes in the major hypomethylated regions of IA3902, however, varied considerably from the other two strains, including: cell division protein FtsA, flagellar basal-body rod protein, glutamine synthetase type I, DNA gyrase subunit A, putative multidrug efflux transporter, putative ferredoxin, selenocysteine synthase, and a hypothetical protein (Supplementary Datasheet 1, Table 7). Flagellar basal-body rod protein (Konkel et al., 2004), DNA gyrase subunit A and putative multidrug efflux transporter (Iovine, 2013), and putative ferredoxin (Van Vliet et al., 2001) are known virulence genes in *Campylobacter*. These and all other genes in the major hypomethylated regions of IA3902 (with the exception of selenocysteine synthase and hypothetical protein) are also associated with virulence in other bacterial species (Klose and Mekalanos, 1997; Ran Kim and Haeng Rhee, 2003; Sjöblom et al., 2008; Kawai et al., 2011; Schweizer, 2012; Swick et al., 2013). The hypomethylation of a large number of genes in a concentrated area of the genome (major hypomethylated region) may suggest a role in gene or virulence expression. However, it remains to be determined whether this is the case and is an indication of gene regulation.

### Minor hypomethylated regions

In addition to the major hypomethylated areas, there were numerous single hypomethylated bins scattered across the genome, which, for ease of description, will be called minor hypomethylated regions. The locations of these regions are described in Supplementary Datasheet 1, Table 6. Genes unique to IA3902 in these areas and listed in Supplementary Datasheet 1, Table 7 included: tetrapyrrole methylase family protein, 16S ribosomal RNA methyltransferase RsmE, phosphoribosylformylglycinamidine synthase I, olylpolyglutamate synthase/dihydrofolate synthase, uracil phosphoribosyltransferase, putative peptide ABC-transport system periplasmic peptide-binding protein, DNA topoisomerase I, putative metallo-beta-lactamase family protein, dimethyladenosine transferase, and several hypothetical proteins. None of these genes are well-known *Campylobacter* virulence factors. However, the genes such as uracil phosphoribosyltransferase (Koyama et al., 2012), putative peptide ABC-transport system periplasmic peptide-binding protein (Garmory and Titball, 2004), DNA topoisomerase I (Galán and Curtiss, 1990), putative metallo-beta-lactamase family protein (Maltezou, 2009), and dimethyladenosine transferase (Chiok et al., 2013) are important for the virulence of other organisms and also serve as potential drug targets. For example, the putative metallo-beta-lactamase family protein found in IA3902 is among a growing list of contributing sources to antimicrobial resistances in ESKAPE pathogens (Bassetti et al., 2013). These antibiotic-resistant pathogens are the cause of a majority of US hospital infections and consist of *E*nterococcus faecium, *S*taphylococcus aureus, *K*lebsiella pneumoniae, *A*cinetobacter baumanii, *P*seudomonas aeruginosa, and *E*nterobacter species (Boucher et al., 2009).

Of the genes unique to 11168 in the minor hypomethylated areas (Supplementary Datasheet 1, Table 7), only fumarate reductase subunits (Kassem et al., 2012), ATP-dependent protease La (Cohn et al., 2007), and molecular chaperone GroEL (Klančnik et al., 2006) were known for their roles in *C. jejuni* virulence. These genes, along with other minor hypomethylated genes (glutamine transporter permease and cystathionine beta-lyase), have virulence homologs in other bacterial organisms (Ejim et al., 2004; Buettner et al., 2008; Zhu et al., 2009; Breidenstein and Hancock, 2012; Kupper et al., 2014). In 81-176, the only genes that were unique to this strain, found in the minor hypomethylated regions, and have a role in *C. jejuni* virulence included RelA/SpoT family protein (Gaynor et al., 2005) and flagellar hook-associated protein FlgK (Fernando et al., 2007; Neal-Mckinney and Konkel, 2012) (Supplementary Datasheet 1, Table 7). These two genes and other genes in the minor hypomethylated regions of 81-176 (succinyl-CoA synthetase (subunits alpha and beta), cell division protein FtsZ, flagellar basal-body rod protein, putative cytochrome oxidase maturation protein (cbb3-type), heavy metal translocating P-type ATPase, uridylate kinase, and 1-deoxy-D-xylulose 5-phosphate reductoisomerase) are also important virulence determinants in other bacterial species (Ji and Silver, 1995; Ran Kim and Haeng Rhee, 2003; Henry et al., 2005; Tchawa Yimga et al., 2006; Wu et al., 2006; Dozot et al., 2006; Jiménez De Bagüés et al., 2007; Lee et al., 2007; Brown and Parish, 2008).

#### Hypermethylation

Even fewer similarities were found in the types of genes that were hypermethylated in all three strains. Genes were considered hypermethylated in a strain if motif frequencies were greater than three standard deviations above the mean: 45 (IA3902) or 41 (11168 and 81-176). IA3902 had two hypermethylated regions, 11168 had one, and 81-176 had four regions. The locations of these hypermethylated regions and the genes present in these regions are shown in Supplementary Datasheet 1, Tables 8, 9, respectively.

The first hypermethylated region in IA3902 was located near the *ori*C and consisted of the following genes (Supplementary Datasheet 1, Table 8): chromosomal replication initiator protein DnaA, glutamate synthase subunit beta, ribonuclease HII, ExsB protein, DsbB family disulfide bond formation protein, methyl-accepting chemotaxis protein, cytochrome c551 peroxidase, FAD-dependent thymidylate synthase, CTP synthetase, and hypothetical proteins. The genes present in the second hypermethylated region toward the end of the genome included putative periplasmic toluene tolerance protein and putative integral membrane protein (Supplementary Datasheet 1, Table 8). Of these genes, only DsbB family disulfide bond formation protein (Łsasica et al., 2010), methyl-accepting chemotaxis protein (Vegge et al., 2009; Li et al., 2014) and cytochrome c551 peroxidase (Hendrixson and Dirita, 2004) are recognized virulence genes in *C. jejuni*. The Dsb protein (Heras et al., 2009), methyl-accepting chemotaxis protein (Dons et al., 2004; Terry et al., 2005; Nishiyama et al., 2012), FAD-dependent thymidylate synthase (Ulmer et al., 2008), and putative periplasmic toluene tolerance protein (Sardessai and Bhosle, 2002) had virulence homologs in other bacterial organisms too. All motifs were found in the hypermethylated genes of IA3902, and RAATTY was detected in every hypermethylated chromosomal gene (Supplementary Datasheet 1, Table 8).

As for the pVir plasmid in IA3902, a number of hypermethylated genes were also found (Supplementary Datasheet 1, Table 9). Such genes included VirB4, VirB8, and VirB9 virulence proteins; phage protein, TrbM-like protein, and numerous hypothetical proteins. Of these genes, VirB9 (Kienesberger et al., 2011) is the only known gene in *Campylobacter* with virulence capabilities. VirB4 (Juhas et al., 2008; Gokulan et al., 2013; Sánchez-Zauco et al., 2013) and VirB8 (Baron, 2006) are well-known for their involvement in Type IV secretion systems, but their virulence roles are unknown in *Campylobacter*. All motifs except for TAAYN_5_TGC were found in the plasmid genes. Only RAATTY was found in most genes, except for CJSA_pVir0050 and CJSA_pVir0051.

The *C. jejuni* strain 11168 had only one hypermethylated gene located in the middle of the genome, with the motifs RAATTY and ACN_5_CTC (Supplementary Datasheet 1, Table 8). This gene, para-aminobenzoate synthase component I, unlike other bacterial organisms (Shinohara et al., 2005), has no known role in *C. jejuni* virulence. 81-176 had four hypermethylated regions: three at the beginning of the genome and one near the end of the genome (Supplementary Datasheet 1, Table 8). The genes present in these regions have no known association with *Campylobacter* virulence (Supplementary Datasheet 1, Table 9). However, RarA has an antimicrobial resistance role in other bacterial species (Veleba et al., 2013; De Majumdar et al., 2013), which, like the other genes not yet found to be involved in *Campylobacter* virulence, could have a potential role for virulence in *C. jejuni*. In addition, all motifs were found in the hypermethylated genes of 81-176 except for TAAYN_5_TGC.

#### General trends in *C. jejuni* strain methylation motifs of hypo- and hypermethylated genes

A common theme among all three strains was that RAATTY was the only motif found in almost every hyper- and hypomethylated gene. The only genes of the genome that did not contain a RAATTY motif were: Flagellar basal-body rod protein (CJSA_0661), putative ferredoxin (CJSA_1311), 16S ribosomal RNAs (Cjr01, Cjr04, CJJ81176_1711, CJJ81176_1724), 23S ribosomal RNAs (Cjr02, Cjr05, Cjr08, CJJ81176_1707, CJJ81176_1727), and putative periplasmic protein (CJJ81176_0993).

Another similarity linking IA3902 with one of the gastroenteric strains was the hypomethylation of the flagellar basal-body rod protein. This was the only hypomethylated gene in common between IA3902 and 81-176, although they were located in different types of hypomethylated regions (major in IA3902, minor in 81-176) (Supplementary Datasheet 1, Table 6). In addition, both strains had the TAAYN_5_TGC motif in the flagellar basal-body rod protein gene.

While there were some similarities among all three strains, we also noticed several differences distinguishing IA3902 from 11168 and 81-176. For instance, IA3902 had a far greater number of genes that were hypo- and hypermethylated in contrast to 11168 and 81-176. This was especially evident with the number of hypermethylated genes in IA3902 chromosome and plasmid surpassing the number of hypermethylated genes in 11168 and 81-176 combined. Eight well-characterized *C. jejuni* virulence genes were found in both the hyper- and hypomethylated regions of IA3902. In contrast, only three or two known *C. jejuni* virulence genes were detected in the hypomethylated regions of 11168 and 81-176, respectively. IA3902 was the only strain to have all its motifs present in the hypermethylated genes. 11168 and 81-176 had at least one motif that was not found in a hypermethylated gene. IA3902 was also the only strain that had hypermethylation at the *ori*C site, specifically in the *dnaA* gene. The significance for hypermethylation at this site in only IA3902 is presently unknown and will require further investigation.

Another difference was the tendency for one motif to dominate over the others in a specific hypomethylated region (major or minor), and only observed in 11168 and 81-176 (Supplementary Datasheet 1, Table 6). For example, in 11168, RAATTY was found most often in the minor hypomethylated areas and GKAAYG was found most often in the major hypomethylated areas. This same pattern was observed in 81-176. Specifically, RAATTY was more prevalent in the minor hypomethylated genes while GGRCA was more prevalent in the major hypomethylated genes. However, for IA3902, the homolog to GKAAYG and GGRCA, GAAGAA, was not the most common motif in the major hypomethylated regions. Instead, it was RAATTY, which is prevalent in the minor as well as the major hypomethylated areas.

The genes in the minor hypomethylated regions (Supplementary Datasheet 1, Table 7) shared only between 11168 and 81-176 included CmeB efflux pump, elongation factor Tu, and Type I restriction enzyme (R subunit). CmeB efflux pump was the only known virulence gene in *C. jejuni* (Lin et al., 2002) among these set of genes while elongation factor Tu has a virulence role in other bacterial species (Kunert et al., 2007; Wang et al., 2013; Al-Maleki et al., 2014; Mohan et al., 2014). The motifs seem to be of the same type when comparing the same gene between 11168 and 81-176 (Supplementary Datasheet 1, Table 6). For example, 11168 had RAATTY and ACN_5_CTC in all three genes while 81-176 had RAATTY and GGRCA in all three of its genes. A trend was observed among this example and the flagellar basal-body rod protein gene present in both IA3902 and 81-176. The pattern observed was that a gene present in two different strains had the same type of motif and was recognized by Type II R-M enzymes. This may indicate conservation in the sequence motif and its associated R-M enzyme for the expression of the particular gene. This conservation may be part of what defines the species or important for the survival of the organism. However, the significance for the conservation of a sequence motif will require further epigenetic studies in other *Campylobacter* species to understand its importance in the evolution of the species.

### Comparative analysis of IA3902 methylated genes by role category with *C. jejuni* gastroenteric strains 11168 and 81-176

The functional roles of the genes were examined based on methylation pattern (hyper- and hypomethylation). Genes were classified into role categories (functional role of the genes) for each motif and all motifs combined. The percent total was then calculated for methylated genes enriched with a specific motif for each role category and made side-by-side comparisons between all three strains (Supplementary Datasheet 1, Table 10).

RAATTY was found in 90-100% of the genes in all role categories for each strain. This corresponded with its prevalence in the hypo- and hypermethylated genes. In addition, RAATTY contained the greatest number of high percentage methylated genes (ranging from 90-100% methylation) in all role categories except for two. These two exceptions were “Mobile and extrachromosomal element functions” and “rRNA and stable RNAs,” which not only had no detectable RAATTY motifs, but also no other motif. The potential reason for not finding motifs in the “rRNA and stable RNAs” category is because modifications on RNA are post-transcriptional (Helm, 2006; Grosjean, 2009). The genes in this category may have motifs on the RNAs recognized by DNA or RNA MTases that may not be detectable by DNA sequencing. Instead, the motifs require other methods for detection such as m6A immunoblotting, m6A-sensitive ligase reaction, and m6A-sensitive reverse transcription or using SMRT technology to reverse transcribe cDNA from RNA templates (Meyer and Jaffrey, 2014).

We also compared role categories between each strain based on motif homology and listed the role categories from highest to lowest percentage in total methylated genes (Supplementary Datasheet 1, Table 11). A few notable differences were found that both distinguished IA3902 from the gastroenteric strains, and from each individual strain. The only categories with genes that were 100% methylated, without regard to a specific motif, and in all three strains were: central intermediary metabolism; DNA metabolism, replication, and repair; regulatory functions, signal transduction, small molecule degradation, and transport and binding proteins. Categories with 100% methylation unique to IA3902 were amino acid biosynthesis and protein fate.

We also found two role categories that either had the highest percent number of genes with a particular motif, or had the lowest or zero percent number of genes with the motif when we compared the three strains. The role category “small molecule degradation” had the highest percent number of genes with the motif GKAAYG (11168) at 100%. On the other hand, the same category for CAAAYG (IA3902) was at 50%. In 81-176, motif GCAAGG was even lower at 20%. This same category also had the highest percent number of genes with the motif GAGN_5_RTG in 11168 (66.67%). However, GAGN_5_RTG was not found in any of the genes in the “small molecule degradation” category for IA3902. Another role category, “pseudogenes/degenerate CDS,” had the highest percentage of genes with the methylated motif GAAGAA at 61.11% for IA3902. The homologous motif in 81-176, GGRCA, was not detected in any of the genes in this category.

### Identification and analyses of MTases not detected in this study

We searched through REBASE to identify what bacterial species had enzymes with the RAATTY recognition sequence. We discovered that 62.5% of the enzymes belonged to *C. jejuni*, which may suggest a particular association between RAATTY and the identity of *C. jejuni*.

This study identified a number of different MTases in IA3902. However, there were several *C. jejuni* MTases with IA3902 homologs not detected in our study. This may have happened because none of the sequence motifs we identified in IA3902 were predicted targets for the MTase homologs described below.

Based on one of the few publications on *C. jejuni* MTases, we found that IA3902 possessed a homolog to the 11168 MTase Cj1461. This DNA MTase was found linked to virulence-related phenotypes including motility, adhesion, and invasion (Kim et al., 2008). The homolog in IA3902 is a putative DNA methylase (CJSA_1385), but no record of CJSA_1385 was found in REBASE. When we ran the sequence in REBASE to find MTase homologs, the closest identity was M.CjeR14ORF8290P. This Type II, site-specific DNA MTase had the highest DNA sequence identity (98%) with CJSA_1385, and is an enzyme isolated from *Campylobacter jejuni* subsp. *jejuni* R14. The closest neighbors to M.CjeR14ORF8290P have motif specificities for 5′-GATC-3′, which suggests the motif recognized by CJSA_1385 is or is similar to GATC. The 81-176 homolog of Cj1461 was CJJ81176_1454, also a Type II site-specific DNA MTase with 98% identity to Cj1461 and CJSA_1385.

Another 11168 MTase that IA3902 had a homolog for was CjeNORF31P (locus tag Cj0031), which is a Type IIS R-M enzyme with 5′-CCCGA-3′/5′-CCCGAA-3′ as its predicted recognition site or motif (Anjum, 2013). It is a phase variable adenine MTase known for its role in coordinated switching of gene expression, in particular adhesion, invasion, biofilm formation capability, and motility (Anjum, 2013). The IA3902 homolog for Cj0031 is CJSA_0032, which is also a Type II R-M enzyme. Though Cj0031 was thought to contain a frameshift in a previous methylome study (Murray et al., 2012), it has subsequently been demonstrated that Cj0031 is a phase variable gene (Anjum, 2013). The phase variable region of Cj0031 contains a poly-G tract of 8-10 guanine nucleotides. Slipped strand mispairing during DNA replication allows for variability in the length of the poly-G tract and thus inducing a frameshift that results in premature termination of the gene. In the case of 11168, phase variable ON isolates have a 9 nucleotide poly-G tract that allows for translation of Cj0031 to continue. In contrast, a poly-G tract length of either 8 or 10 will result in termination of the translation and the loss of function of this MTase. The 11168 isolate that was sequenced in the original methylome paper was evidently a phase OFF variant that resulted in a non-active MTase (Anjum, 2013).

When we used BLAST to compare Cj0031 with the corresponding CJSA_0032 gene, we found the poly-G tract was gone and the 3′ end of the gene was replaced with a non-phase variable 1130 bp sequence in IA3902. This replacement resulted in a constitutive “phase ON” MTase in contrast to 11168. Furthermore, by using BLAST to compare the non-phase variable region of CJSA_0032 with other *Campylobacter* strains we found four strains with the exact same or similar sequences that were also identified as a Type II R-M enzyme. *C. jejuni* subsp. *jejuni* ICDCCJ07001 and *C. coli* RM1875 had the highest percent match with CJSA_0032 at a value of 97% (sequences from both strains were 1130 bp long). *C. jejuni* subsp. *doylei* 269.97 had 89% homology with a sequence length of 1119 bp. Lastly, *C. lari* RM2100 had 85% homology with a sequence length of 1126 bp.

Two genes coding for RNA MTases in 11168 (Cj0588 and Cj0693c) were also identified. BLAST analysis revealed that at least 99% of these two genes' sequences were homologous with genes in IA3902 (CJSA_0556 and CJSA_0657, respectively). A potential reason for why our study could not detect these two RNA MTases in IA3902 may be due to the inability for the DNA sequencing aspect of SMRT technology to detect such MTases. However, other methods could be performed to detect the RNA MTases as described earlier in the results (Meyer and Jaffrey, 2014).

### Comparative methylome analysis of IA3902 wildtype and *luxS* mutant

**Figure 5:**
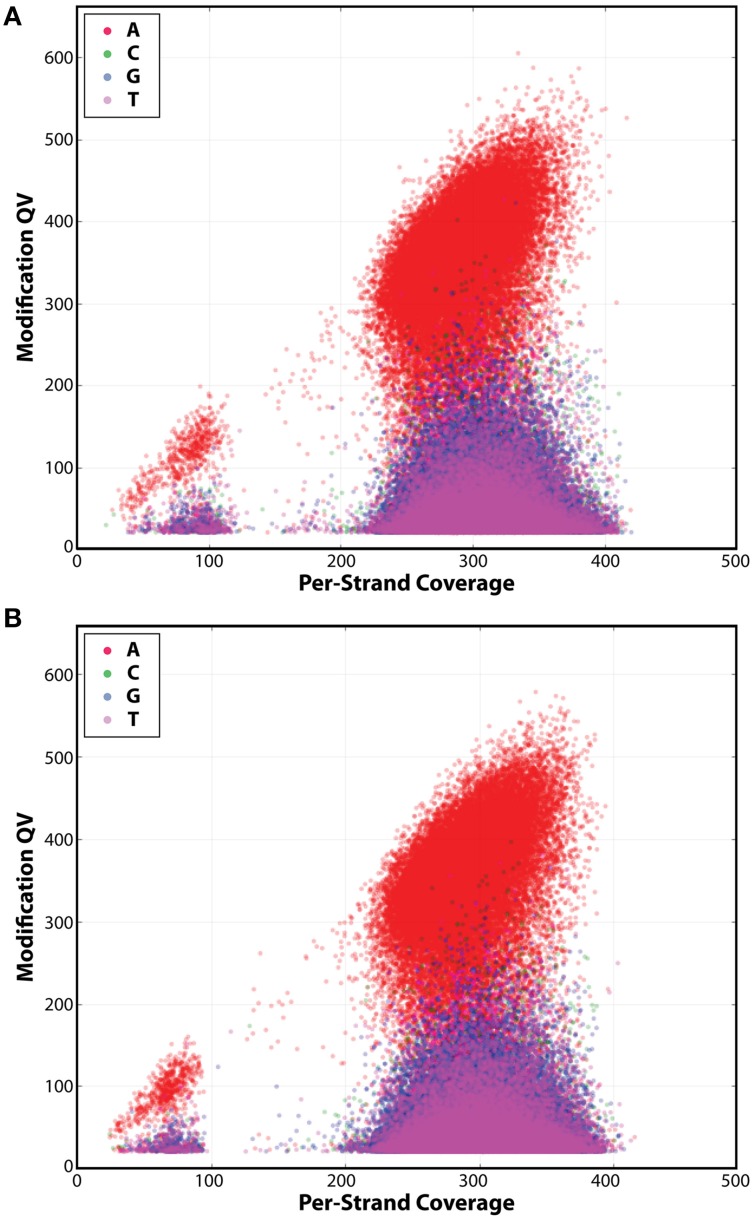
**Detection of N6-methyladenine (m6A) base modification in both IA3902 strains**. The following kinetic score distributions show significantly higher numbers of adenine residues above the background in IA3902 wildtype **(A)** and *luxS* mutant **(B)**. This indicates that the only type of base modification in the wildtype and *luxS* mutant is m6A.

SMRT sequencing was also carried out on the *luxS* mutant of IA3902 and it also yielded one circular chromosome and plasmid. Mutagenesis of the *luxS* gene was also confirmed in the *luxS* mutant strain when we compared its genome sequence with that of the wild-type strain. Sequencing analyses of the methylation sites (Table 3) and base modification type (Figure 5) revealed very little differences between the two genomes. The *luxS* mutant possessed the same seven total sequence motifs as the wildtype (Table 3) and more than 99% of the motifs had the m6A base modification (Table 3, Figure 5). Since results showed the methylation types and patterns were the same between both the wild type and the *luxS* mutant strains, this suggests that LuxS did not have any effect on the IA3902 methylation phenotype when grown in Mueller–Hinton broth.

**Table 3:** **Methylome motifs detected within the *C. jejuni* IA3902Δ*luxS* genome**.

**Motif^a^**	**Modification type**	**# Of motifs detected**	**# Of motifs in genome**	**% Motifs detected**	**Partner motif**	**R-M enzymes^d^^,^^e^**
GAGNNNNNRTG^b^	m6A	717	718	99.86	CAYNNNNNCTC	CjeIAORF994P
CAYNNNNNCTC^b^	m6A	716	718	99.72	GAGNNNNNRTG	CjeIAORF994P
CAAAYG	m6A	1761	1761	100		CjeIAORF654P
TAAYNNNNNTGC^c^	m6A	499	499	100	GCANNNNNRTTA	M.CjeIAII
GCANNNNNRTTA^c^	m6A	494	499	99	TAAYNNNNNTGC	M.CjeIAII
GAAGAA	m6A	2559	2564	99.8		CjeIAORF32P
RAATTY	m6A	27298	27518	99.2	RAATTY	M.CjeIAI

a *Sequence motifs are listed in the 5 ′ to 3 ′ direction. Underlined bases indicate the methylated base on the sequence. Motifs containing Y have either T or C nucleotide, while motifs with R have an A or G nucleotide*.

b,c *Complementary motif sequences*.

d *Last column lists assigned restriction-modification (R-M) enzymes predicted to recognize respective motif(s)*.

e *The motifs and associated MTases are excellent candidates for the respective R-M system types. However, because the designations are not definitive, further tests are required to confirm the functional statuses of the MTases*.

## Discussion

When we compared the methylation profile of IA3902 with 11168 and 81-176, there were both similarities and differences between their methylation patterns. A majority of their motif sequences and MTases were homologous (Supplementary Datasheet 1, Tables 1–5), with one or two motifs and MTases without an IA3902 homolog in 11168 and 81-176. The methylation motif distribution plot (Figures 1–4) and the number and types of genes within the hypo- and hypermethylated regions (Supplementary Datasheet 1, Tables 7, 9) were vastly different between IA3902 and the other two *C. jejuni* strains. For example, a higher number of hyper- and hypomethylated genes were found in IA3902 in contrast to the other two *C. jejuni* strains. IA3902 also had a higher proportion of well-characterized *C. jejuni* virulence genes than 11168 and 81-176 individually and combined. This suggests that restriction and modification activities may play a stronger role in the expression of IA3902 genes (including virulence genes) more so than the gastroenteric strains. The high proportion of hyper- and hypomethylated virulence genes in IA3902 may thus be correlated with the strain's hyper virulence and abortion-causing phenotype.

Most of the genes in the major hypomethylated region of 11168 and 81-176 were 16S and 23S rRNA genes (Supplementary Datasheet 1, Table 7). The reason for this is unclear, and to our knowledge similar findings of hypomethylation of ribosomal genes in other bacteria have not been described. Interestingly, MTases can also modify ribosomal RNA genes by post-transcriptional methylation and are most well-characterized in *Escherichia coli* (Baldridge and Contreras, 2014). Several studies have found that knocking out the MTase gene conferred an increased level of antibiotic resistance (Lamarre et al., 2011; Mikheil et al., 2012; Monshupanee et al., 2012; Sałamaszyñska-Guz et al., 2014). These findings, combined with our identification of these genome regions as hypomethylated, suggest that further studies are warranted to determine the physiologic rationale for the hypomethylation of these rRNA genes.

It is interesting that all three *C. jejuni* strains had such a high number of and multiple types of R-M systems for its small genome size (Vasu and Nagaraja, 2013). RAATTY was also the most prevalent motif of almost every hyper- and hypomethylated gene in all three strains. The prevalence of RAATTY and presence of so many R-M systems may be attributed to one or several reasons. First, they may serve to help stabilize the host chromosome. R-M systems parallel toxin-antitoxin systems, which are abundant and help to stabilize neighboring chromosomal regions of the genome (Mruk and Kobayashi, 2013). Especially with the small genome size of *C. jejuni* and natural transformative ability of the organism, R-M systems (along with the high prevalence of RAATTY motifs) can help to maintain genomic islands acquired during horizontal gene transfer events.

Second, R-M systems and the RAATTY motif may help to genetically isolate IA3902 from 11168 and 81-176, particularly as the strains have different tissue tropism and disease presentations. Enforced methylation is a common phenomenon in Type I, II and IV R-M systems (Ishikawa et al., 2010). Enzymes in these systems cause cell death when the host bacteria exhibits altered methylation patterns, thus ensuring the epigenetic status of the population. With the small genome size of *C. jejuni*, perhaps the Type I and II R-M systems help to protect the methylation pattern of the host genome and prevent any harmful changes that would threaten its survival. It is also interesting that all three *C. jejuni* strains possess the m6A form of base modification. The numerous MTases and associated R-M systems that specifically form m6A modifications have diverse roles in cell maintenance and virulence (Wion and Casadesus, 2006; Low and Casadesús, 2008). It is thus logical that *C. jejuni* possess so many MTases and R-M systems to carry out as many functions for its small genome and limited number of genes. RAATTY may also help to maintain the identity of *C. jejuni*. We searched through REBASE to identify what bacterial species had enzymes with the RAATTY recognition sequence. We discovered that 62.5% of the enzymes belonged to *C. jejuni*, which may suggest a particular association between RAATTY and the identity of *C. jejuni*.

As for the polyG tract in Cj0031 of 11168, it is predicted to be replaced with an 1130 bp sequence in CJSA_0032 of IA3902 through a horizontal gene transfer event, while the beginning and end regions of the two genes remained the same. In addition, based on sequence homology, we believe the 1130 bp sequence was likely acquired from one of several *Campylobacter* strains. It is a well-known phenomenon for R-M systems to undergo horizontal gene transfer events (Sharp et al., 1992; Nobusato et al., 2000; Bujnicki, 2001). In addition, such events can occur with DNA fragments as large as the 1130 bp fragment found in this study (Aras et al., 2002). R-M systems are even referred to as “selfish mobile elements” with the sole purpose of promoting its survival (Kobayashi, 2001), which may explain the phase ON state of CJSA_0032.

One possible explanation for the selection of the phase ON state is environmental pressures favoring this state. The possession of a phase variable MTase can generate multiple cell types with distinct virulence expression profiles via global changes in methylation (Srikhanta et al., 2005; Krebes et al., 2014). This was evident in the study (Anjum, 2013) when phase ON variants of Cj0031 in 11168 were selected over phase OFF after passage *in vivo* in a chicken host. It was predicted that phase ON Cj0031 allowed the MTase to regulate expression of other genes required for host adaptation. Thus it can be predicted that the phase ON state may enable IA3902 to thrive in its specific niche and generate its abortifacient virulence profile.

The unique methylation patterns as a result of the phase ON state of CJSA_0032 may have caused the formation of IA3902 in becoming a new “biotype” (Vasu and Nagaraja, 2013) or a different variant of 11168, with a distinct identity from 11168 and other *C. jejuni* strains. Phase variable R-M systems can control the uptake of foreign DNA, thus serving as both a defense mechanism as well as regulating the influx of DNA. It may be that IA3902 lost the phase variable function in CJSA_0032 as a way to prevent suicidal restriction of its DNA, or that the function was redundant (Fox et al., 2007).

The results from this study have provided new insights for understanding the impact of methylation on *C. jejuni* virulence and evolution. Despite the emergence of sequencing technologies to detect methylation in prokaryotic genomes, very few studies have looked at the significance of methylation in genes such as the ones identified in this study. Though aspects of this study have revealed some clues, there were also major limitations that added challenges and complexities to this study. One limitation to this study is that the methylome analysis is based on strains grown in basal conditions. Additional work is needed to develop a more complete appreciation of the role of culture conditions in the methylomes of these strains. Using different culture conditions that would enable changes in gene expression would also provide a better understanding of what genes under what conditions have their expression regulated by methylation. Another limitation of this study is that the number of passages and differences in time of isolation between the strains could result in changes to the methylome. This assertion is, to our knowledge, true of most comparative genomic studies that contain strains from varying sources, period of disease, and differing number of passages. However, findings from this study still hold value for developing new hypotheses and provide the first information regarding the role of the methylome in these strains. The inability to include a large number of replicate strains in this study was a third limitation. Methylation-calling in SMRT sequencing requires greater sequencing depth than that for base-calling alone. The inclusion of the IA3902 *luxS* mutant strain provided a replicate and demonstrated the repeatability of the process while also providing validity to the results presented. Since both strains demonstrated identical methylomes, additional replicates were out of the scope of this study. In addition to the study limitations, much remains to be explored in terms of the significance of methylation (hypermethylation vs. hypomethylation) on the genes examined in this study.

While work is ongoing to gain insight into the functional roles of methylation in *C. jejuni* virulence expression, several studies have already found potential roles for methylation in regulating the expression of genes, including those associated with virulence. In *E. coli*, MTases were associated with increased expression of cation transport as well as decreased expression in cell projection, flagellar motility, and flagellum (Fang et al., 2012). Similar to the hypermethylation observed in IA3902 at the *oriC* region, hypermethylation at the *oriC* site was discovered in *Shewanella oneidensis* (Bendall et al., 2013). The authors from the *S. oneidensis* study paralleled this phenomenon with what has been observed in *E. coli* and suggest this hypermethylation may have a role in regulating genome replication. In *Mycoplasma pneumoniae*, hypermethylation was observed in two functional gene groups (defense mechanisms and genes coding for membrane proteins or lipoproteins) and one of its main virulence factors (Lluch-Senar et al., 2013). Hypermethylation was also discovered in regions containing putative DnaA boxes, suggesting that DNA methylation may play a role in DNA replication in *M. pneumoniae*.

Several studies have also explored the impact of phase variable MTases on gene expression in other bacterial species. In *Neisseria* sp., it was found that changing methylation patterns and altered gene expression by its phase variable MTases gives the organism opportunities to adapt in the host (Srikhanta et al., 2009). The phase variable MTase activity and random switching of virulence factors expression were also tested in *Haemophilus influenzae* (Srikhanta et al., 2005). MTases were found to have enhanced expression of several types of genes that enabled improved fitness of *H. influenzae* to environmental and physiological stresses. The findings from these methylation studies give indication that methylation (hyper- and hypomethylation) of genes observed in our *C. jejuni* strains, including virulence genes, and the MTases identified in this study could be regulating the expression of these genes.

Earlier, it was discussed how R-M systems serve to maintain the species of a bacteria (Vasu and Nagaraja, 2013). We are also greatly interested in determining whether a species and its virulence are defined by the R-M systems present in its genome. Moreover, how would the MTase specific to one species behave in another species? This also brings to question if methylation is a key evolutionary event for other *Campylobacter* species as it may be for IA3902, 11168, and 81-176. In practical applications, uncovering the mechanisms for methylation on *Campylobacter* gene expression would make DNA methylation a promising drug target against diseases of *Campylobacter* and related organisms, as has been exploited in other bacteria (Mashhoon et al., 2004; Feder et al., 2008; Mckelvie et al., 2013).

In terms of the impact of the *luxS* mutation on the methylation patterns of IA3902, we found no significant differences between the *luxS* mutant and wildtype. Both strains' genomes only displayed the m6A base modification (Figure 5). In addition, the seven motifs, along with the MTases recognizing the motifs, were also the same for both strains (Tables 1, 3). We conclude that the mutation in the *luxS* gene had no appreciable effect on the genome-wide methylation on IA3902. This suggests that a non-functioning *luxS* gene had no effect on the methylation profile of IA3902, and thus cannot explain for the attenuated virulence of IA3902Δ*luxS* (Plummer et al., 2012).

There may be several reasons why no differences were observed in the methylation patterns between IA390 and IA3902Δ*luxS*. One hypothesis is that methylation may have been affected but because an exogenous source of methionine was provided (e.g., the Mueller-Hinton media it was grown in), this allowed the organism to preserve its methylation status. Or, the *luxS* mutant employed an alternative mechanism to bypass the step requiring LuxS enzyme to complete the AMC cycle. The mutant could have also substituted the AMC cycle with another pathway to generate methyl groups and complete methylation. Although there is no proof for these hypotheses, they warrant further study. Despite the lack of differences found between IA3902 and its *luxS* mutant, we obtained novel information about the motifs and MTases of IA3902. For example, this study revealed two novel motifs, CAAAYG and GAAGAA, and their putative assignments to R-M enzymes CjeIAORF654P and CjeIAORF32P, respectively.

In conclusion, the methylation patterns of IA3902 were not affected by a non-functional *luxS* gene. In addition, though the motif sequences and MTases between IA3902 and the gastroenteric 11168 and 81-176 strains were similar, their methylation profiles (including types of genes methylated and the hypo- and hypermethylation regions in the methylation motif distribution plots) were very different and enriched with known virulence genes. The results from this study have raised many questions regarding the impacts of methylation in *C. jejuni* virulence. However, these findings also revealed the immense potential methylation plays in *Campylobacter* pathobiology. The impact from this will not only help determine the disease pathogenicity of this and other organisms, but also serve as a strong candidate for developing novel therapeutics against *C. jejuni* diseases.

## Author contributions

Kathy T. Mou and Paul J. Plummer designed the study. Tyson A. Clark and Matthew Boitano performed SMRT sequencing and generated methylation data. Kathy T. Mou, Usha K. Muppirala, Andrew J. Severin, and Paul J. Plummer analyzed methylation data. Kathy T. Mou and Paul J. Plummer prepared the manuscript. Usha K. Muppirala, Andrew J. Severin, Tyson A. Clark, and Matthew Boitano gave technical support, conceptual advice, and edited the manuscript.

### Conflict of interest statement

Tyson A. Clark and Matthew Boitano are full time employees of Pacific Biosciences, a company commercializing SMRT sequencing technologies. Pacific Biosciences provided the PacBio sequencing and some of the sequence assembly and analysis free of charge. The authors declare that the research was conducted in the absence of any commercial or financial relationships that could be construed as a potential conflict of interest.

## Abbreviations

m6A: N6-methyladenine
R-M: Restriction-modification
REBASE: Restriction Enzyme Database
SA: Sheep Abortion
SMRT: Single Molecule, Real-Time.
