# Supplementary material for: A comparative analysis of methylome profiles of Campylobacter jejuni sheep abortion isolate and gastroenteric strains using PacBio data
Source: Front Microbiol. 2015 Jan 14;5:782. doi: 10.3389/fmicb.2014.00782 (PMC4294202; doi:10.3389/fmicb.2014.00782)
Supplement: Supplementary file 1 [file DataSheet1.DOCX]

***Supplementary Material***

**A comparative methylome analysis of *Campylobacter jejuni* abortifacient isolate IA3902 to syntenic strains of gastroenteric origin and the impact of LuxS mutagenesis on the methylome of IA3902**

**Kathy T. Mou^1^, Usha K. Muppirala^2^, Andrew J. Severin^2^, Tyson A. Clark^3^, Matthew Boitano^3^, and Paul J. Plummer^4*^**

^1^Department of Veterinary Microbiology & Preventive Medicine, College of Veterinary Medicine, Iowa State University, Ames, Iowa, USA

^2^Genome Informatics Facility, Office of Biotechnology, Iowa State University, Ames, IA, USA

^3^Pacific Biosciences, Menlo Park, California, USA

^4^Department of Veterinary Diagnostic and Production Animal Medicine, College of Veterinary Medicine, Iowa State University, Ames, Iowa, USA

***Correspondence:** Paul Plummer, Iowa State University, College of Veterinary Medicine, Department of Veterinary Diagnostic and Production Animal Medicine, 2426 Lloyd Vet Med Ctr., Ames, IA, 50011, USA.
pplummer@iastate.edu

1. **Supplementary Data**
   1. Supplementary Data 1-4: Motif and methylation scores
      1. Supplementary Data 1 *C. jejuni* IA3902 wildtype motif and methylation scores.xlsx
      2. Supplementary Data 2 *C. jejuni* IA3902 luxS mutant motif and methylation scores.xlsx
      3. Supplementary Data 3 *C. jejuni* 11168 motif and methylation scores.xlsx
      4. Supplementary Data 4 *C. jejuni* 81-176 motif and methylation scores.xlsx
2. **Supplementary Tables**
   1. **Supplementary Table 1. Motif comparisons between IA3902, 11168 and 81-176 grouped by IA3902 motifs TAAYNNNNNTGC and GCANNNNNRTTA.**
      1. ^a^Modified bases of each motif are underlined.

| **Strain** | **Motif^a^** | **Enzyme (Locus tag)** | **Gene description** | **Number of the motif in genome** | **Number of the motif detected** | **% motif detected** | **% DNA homology with IA3902 MTase** |
| --- | --- | --- | --- | --- | --- | --- | --- |
| IA3902 | 5’-TAAYNNNNNTGC-3’ | M.CjeIAII (CJSA_1469) | Putative Type I R-M enzyme, M subunit | 499 | 499 | 100 | X |
|  | 3’-ATTRNNNNNACG-5’ |  |  | 499 | 493 | 98.8 |  |
| 11168 | 5’-TAAYNNNNNTGC-3’ | M.CjeNIV (Cj1553c) | Type I R-M system, M subunit | 469 | 452 | 96.4 | 100 |
|  | 3’-ATTRNNNNNACG-5’ |  |  | 469 | 454 | 96.8 |  |
| 81-176 | 5’-TAAYNNNNNTGC-3’ | M.CjeFIV (CJJ81176_1539) | Type I R-M system, M subunit | 460 | 441 | 95.9 | 100 |
|  | 3’-ATTRNNNNNACG-5’ |  |  | 460 | 449 | 97.6 |  |

- 1. **Supplementary Table 2. Motif comparisons between IA3902, 11168 and 81-176 grouped by IA3902 motif RAATTY.**
     1. ^a^Modified bases of each motif are underlined.

| **Strain** | **Motif^a^** | **Enzyme (Locus tag)** | **Gene description** | **Number of the motif in genome** | **Number of the motif detected** | **% motif detected** | **% DNA homology with IA3902 MTase** |
| --- | --- | --- | --- | --- | --- | --- | --- |
| IA3902 | 5’-RAATTY-3’ | M.CjeIAI (CJSA_0199) | Type II D12 class N6 adenine-specific DNA methyltransferase | 27,514 | 27,318 | 99.2 | X |
|  | 3’-YTTAAR-5’ |  |  |  |  |  |  |
| 11168 | 5’-RAATTY-3’ | M.CjeNI (Cj0208) | Type II DNA modification methylase | 27,080 | 27,056 | 99.2 | 100 |
|  | 3’-YTTAAR-5’ |  |  |  |  |  |  |
| 81-176 | 5’-RAATTY-3’ | M.CjeFI (CJJ81176_0240) | Type II D12 class N6 adenine-specific DNA methyltransferase | 26,876 | 26,609 | 99 | 100 |
|  | 3’-YTTAAR-5’ |  |  |  |  |  |  |

- 1. **Supplementary Table 3. Motif comparisons between IA3902, 11168 and 81-176 grouped by IA3902 motif CAAAYG.**
     1. ^a^Modified bases of each motif are underlined.

| **Strain** | **Motif^a^** | **Enzyme (Locus tag)** | **Gene description** | **Number of the motif in genome** | **Number of the motif detected** | **% motif detected** | **% DNA homology with IA3902 MTase** |
| --- | --- | --- | --- | --- | --- | --- | --- |
| IA3902 | 5’-CAAAYG-3’ | CjeIAORF654P (CJSA_0654) | Putative Type II R-M enzyme | 1760 | 1760 | 100 | X |
|  | 3’-GTTTRC-5’ |  |  |  |  |  |  |
| 11168 | 5’-GKAAYG-3’ | CjeNIII (Cj0690c) | Type II R-M enzyme | 1259 | 1236 | 98.2 | 96 |
|  | 3’-CMTTRC-5’ |  |  |  |  |  |  |
| 81-176 | 5’-GCAAGG-3’ | CjeFIII (CJJ81176_0713) | Type II R-M, hypothetical protein | N/A | N/A | N/A | 97 |
|  | 3’-CGTTCC-5’ |  |  |  |  |  |  |

- 1. **Supplementary Table 4. Motif comparisons between IA3902, 11168 and 81-176 grouped by IA3902 motifs GAGNNNNNRTG and CAYNNNNNCTC.**
     1. ^a^Modified bases of each motif are underlined.

| **Strain** | **Motif^a^** | **Enzyme (Locus tag)** | **Gene description** | **Number of the motif in genome** | **Number of the motif detected** | **% motif detected** | **% DNA homology with IA3902 MTase** |
| --- | --- | --- | --- | --- | --- | --- | --- |
| IA3902 | 5’-GAGNNNNNRTG-3’ | CjeIAORF994P (CJSA_0994) | Type II R-M enzyme | 717 | 717 | 100 | X |
|  | 3’-CTCNNNNNYAC-5’ |  |  | 717 | 716 | 99.8 |  |
| 11168 | 5’-GAGNNNNNGT-3’ | CjeNII (Cj1051c) | Type II R-M enzyme | 1066 | 1051 | 98.6 | 86 |
|  | 3’-CTCNNNNNCA-5’ |  |  | 1066 | 1053 | 98.8 |  |

- 1. **Supplementary Table 5. Motif comparisons between IA3902, 11168 and 81-176 grouped by IA3902 motif GAAGAA.**
     1. ^a^Modified bases of each motif are underlined.

| **Strain** | **Motif^a^** | **Enzyme (Locus tag)** | **Gene description** | **Number of the motif in genome** | **Number of the motif detected** | **% motif detected** | **% DNA homology with IA3902 MTase** |
| --- | --- | --- | --- | --- | --- | --- | --- |
| IA3902 | 5’-GAAGAA-3’ | CjeIAORF32P (CJSA_0032) | Type II R-M enzyme | 2563 | 2557 | 99.7 | X |
|  | 3’-CTTCTT-5’ |  |  |  |  |  |  |
| 81-176 | 5’-GGRCA-3’ | CjeFV (CJJ81176_0713) | Type II R-M, hypothetical protein | 1849 | 1805 | 97.6 | 65 |
|  | 3’-CCYGT-5’ |  |  |  |  |  |  |

- 1. **Supplementary Table 6. Motifs and associated hypomethylated genes detected in IA3902, 11168, and 81-176.***
     1. ***Bolded genes** are found in major hypomethylated areas

| Strain | Motif | Gene name | Locus tag | Gene description | Start Position | End Position |
| --- | --- | --- | --- | --- | --- | --- |
| IA3902 | TAAYNNNNNTGC | **flgG2** | **CJSA_0661** | **flagellar basal-body rod protein** | **658855** | **659667** |
|  |  | **CJSA_1309** | **CJSA_1309** | **putative multidrug efflux transporter** | **1317789** | **1319084** |
|  |  | CJSA_1619 | CJSA_1619 | putative metallo-beta-lactamase family protein | 1619090 | 1621078 |
|  |  | ksgA | CJSA_1620 | dimethyladenosine transferase | 1621053 | 1621853 |
|  | GCANNNNNRTTA | **gyrA** | **CJSA_0970** | **DNA gyrase subunit A** | **960498** | **963089** |
|  |  | **CJSA_1311** | **CJSA_1311** | **putative ferredoxin** | **1319883** | **1321544** |
|  |  | CJSA_1619 | CJSA_1619 | putative metallo-beta-lactamase family protein | 1619090 | 1621078 |
|  |  | CJSA_1621 | CJSA_1621 | hypothetical protein | 1621935 | 1622471 |
|  | RAATTY | CJSA_0145 | CJSA_0145 | tetrapyrrole methylase family protein | 157306 | 158130 |
|  |  | CJSA_0147 | CJSA_0147 | 16S ribosomal RNA methyltransferase RsmE | 158412 | 159068 |
|  |  | purQ | CJSA_0484 | phosphoribosylformylglycinamidine synthase I | 484720 | 485367 |
|  |  | CJSA_0485 | CJSA_0485 | hypothetical protein | 485364 | 486560 |
|  |  | **CJSA_0658** | **CJSA_0658** | **hypothetical protein** | **654696** | **656186** |
|  |  | **ftsA** | **CJSA_0659** | **cell division protein FtsA** | **656189** | **657571** |
|  |  | **glnA** | **CJSA_0663** | **glutamine synthetase, type I** | **660554** | **661984** |
|  |  | **gyrA** | **CJSA_0970** | **DNA gyrase subunit A** | **960498** | **963089** |
|  |  | folC | CJSA_1030 | olylpolyglutamate synthase/dihydrofolate synthase | 1022729 | 1023901 |
|  |  | CJSA_1031 | CJSA_1031 | hypothetical protein | 1023879 | 1025276 |
|  |  | CJSA_1223 | CJSA_1223 | hypothetical protein | 1221784 | 1222455 |
|  |  | upp | CJSA_1224 | uracil phosphoribosyltransferase | 1222461 | 1223087 |
|  |  | **CJSA_1309** | **CJSA_1309** | **putative multidrug efflux transporter** | **1317789** | **1319084** |
|  |  | **selA** | **CJSA_1312** | **selenocysteine synthase** | **1321622** | **1322944** |
|  |  | CJSA_1496 | CJSA_1496 | putative peptide ABC-transport system periplasmic peptide-binding protein | 1510393 | 1511928 |
|  |  | topA | CJSA_1595 | DNA topoisomerase I | 1604754 | 1606856 |
|  |  | CJSA_1619 | CJSA_1619 | putative metallo-beta-lactamase family protein | 1619090 | 1621078 |
|  |  | ksgA | CJSA_1620 | dimethyladenosine transferase | 1621053 | 1621853 |
|  |  | CJSA_1621 | CJSA_1621 | hypothetical protein | 1621935 | 1622471 |
|  | CAAAYG | **CJSA_0658** | **CJSA_0658** | **hypothetical protein** | **654696** | **656186** |
|  |  | CJSA_1223 | CJSA_1223 | hypothetical protein | 1221784 | 1222455 |
|  |  | CJSA_1496 | CJSA_1496 | putative peptide ABC-transport system periplasmic peptide-binding protein | 1510393 | 1511928 |
|  |  | CJSA_1619 | CJSA_1619 | putative metallo-beta-lactamase family protein | 1619090 | 1621078 |
|  | CAYNNNNNCTC | **CJSA_0658** | **CJSA_0658** | **hypothetical protein** | **654696** | **656186** |
|  |  | folC | CJSA_1030 | olylpolyglutamate synthase/dihydrofolate synthase | 1022729 | 1023901 |
|  |  | CJSA_1619 | CJSA_1619 | putative metallo-beta-lactamase family protein | 1619090 | 1621078 |
|  | GAGNNNNNRTG | none |  |  |  |  |
|  | GAAGAA | CJSA_0145 | CJSA_0145 | tetrapyrrole methylase family protein | 157306 | 158130 |
|  |  | **glnA** | **CJSA_0663** | **glutamine synthetase, type I** | **660554** | **661984** |
|  |  | **gyrA** | **CJSA_0970** | **DNA gyrase subunit A** | **960498** | **963089** |
|  |  | **CJSA_1311** | **CJSA_1311** | **putative ferredoxin** | **1319883** | **1321544** |
|  |  | CJSA_1496 | CJSA_1496 | putative peptide ABC-transport system periplasmic peptide-binding protein | 1510393 | 1511928 |
|  |  | topA | CJSA_1595 | DNA topoisomerase I | 1604754 | 1606856 |
|  |  | CJSA_1621 | CJSA_1621 | hypothetical protein | 1621935 | 1622471 |
| 11168 | TAAYNNNNNTGC | mloB | Cj1552c | hypothetical protein | 1485626 | 1487092 |
|  | GCANNNNNRTTA | **Cjr08** | **Cjr08** | **23S ribosomal RNA** | **698743** | **701654** |
|  |  | Cj1389 | Cj1389 | pseudo | 1325668 | 1327107 |
|  | RAATTY | cmeB | Cj0366c | inner membrane efflux transporter CmeB | 332596 | 335718 |
|  |  | frdC | Cj0408 | fumarate reductase cytochrome b-556 subunit | 372589 | 373371 |
|  |  | frdA | Cj0409 | fumarate reductase flavoprotein subunit | 373355 | 375346 |
|  |  | tuf | Cj0470 | elongation factor Tu | 434408 | 435607 |
|  |  | **Cjr07** | **Cjr07** | **16S ribosomal RNA** | **696424** | **697936** |
|  |  | Cj0939c | Cj0939c | hypothetical protein | 877647 | 878027 |
|  |  | glnP | Cj0940c | glutamine transporter permease | 878828 | 879613 |
|  |  | ssb | Cj1071 | single-stranded DNA-binding protein | 1005538 | 1006089 |
|  |  | rpsR | Cj1072 | 30S ribosomal protein S18 | 1006100 | 1006360 |
|  |  | lon | Cj1073c | ATP-dependent protease La | 1006421 | 1008796 |
|  |  | groEL | Cj1221 | molecular chaperone GroEL | 1149475 | 1151112 |
|  |  | Cj1389 | Cj1389 | pseudo | 1325668 | 1327107 |
|  |  | metC | Cj1393 | cystathionine beta-lyase | 1327236 | 1328282 |
|  |  | hsdR | Cj1549c | type I restriction enzyme R protein | 1479390 | 1482485 |
|  |  | mloB | Cj1552c | hypothetical protein | 1485626 | 1487092 |
|  |  | hsdM | Cj1553c | type I restriction enzyme M protein | 1487089 | 1488591 |
|  | GKAAYG | **Cjr01** | **Cjr01** | **16S ribosomal RNA** | **39249** | **40761** |
|  |  | **Cjr02** | **Cjr02** | **23S ribosomal RNA** | **41568** | **44457** |
|  |  | cmeB | Cj0366c | inner membrane efflux transporter CmeB | 332596 | 335718 |
|  |  | **Cjr05** | **Cjr05** | **23S ribosomal RNA** | **396449** | **399360** |
|  |  | **Cjr07** | **Cjr07** | **16S ribosomal RNA** | **696424** | **697936** |
|  |  | **Cjr08** | **Cjr08** | **23S ribosomal RNA** | **698743** | **701654** |
|  |  | Cj0939c | Cj0939c | hypothetical protein | 877647 | 878027 |
|  |  | lon | Cj1073c | ATP-dependent protease La | 1006421 | 1008796 |
|  |  | groEL | Cj1221 | molecular chaperone GroEL | 1149475 | 1151112 |
|  |  | Cj1389 | Cj1389 | pseudo | 1325668 | 1327107 |
|  |  | hsdR | Cj1549c | type I restriction enzyme R protein | 1479390 | 1482485 |
|  |  | mloB | Cj1552c | hypothetical protein | 1485626 | 1487092 |
|  | GAGNNNNNGT | cmeB | Cj0366c | inner membrane efflux transporter CmeB | 332596 | 335718 |
|  |  | frdC | Cj0408 | fumarate reductase cytochrome b-556 subunit | 372589 | 373371 |
|  |  | frdA | Cj0409 | fumarate reductase flavoprotein subunit | 373355 | 375346 |
|  |  | tuf | Cj0470 | elongation factor Tu | 434408 | 435607 |
|  |  | **Cjr07** | **Cjr07** | **16S ribosomal RNA** | **696424** | **697936** |
|  |  | hsdR | Cj1549c | type I restriction enzyme R protein | 1479390 | 1482485 |
|  | ACNNNNNCTC | cmeB | Cj0366c | inner membrane efflux transporter CmeB | 332596 | 335718 |
|  |  | tuf | Cj0470 | elongation factor Tu | 434408 | 435607 |
|  |  | hsdM | Cj1553c | type I restriction enzyme M protein | 1487089 | 1488591 |
|  | GAGANNNNGMT | **Cjr01** | **Cjr01** | **16S ribosomal RNA** | **39249** | **40761** |
|  |  | frdA | Cj0409 | fumarate reductase flavoprotein subunit | 373355 | 375346 |
|  |  | **Cjr04** | **Cjr04** | **16S ribosomal RNA** | **394130** | **395642** |
|  |  | **Cjr07** | **Cjr07** | **16S ribosomal RNA** | **696424** | **697936** |
|  |  | mloB | Cj1552c | hypothetical protein | 1485626 | 1487092 |
| 81-176 | TAAYNNNNNTGC | **rrlA** | **CJJ81176_1707** | **23S ribosomal RNA** | **41513** | **44306** |
|  |  | ftsZ | CJJ81176_0719 | cell division protein FtsZ | 649668 | 650780 |
|  |  | CJJ81176_0720 | CJJ81176_0720 | flagellar basal-body rod protein | 650936 | 651748 |
|  |  | CJJ81176_1459 | CJJ81176_1459 | flagellar hook-associated protein FlgK | 1378700 | 1380526 |
|  | GCANNNNNRTTA | CJJ81176_1743 | CJJ81176_1743 | putative periplasmic protein | 913709 | 915370 |
|  | RAATTY | **CJJ81176_0061** | **CJJ81176_0061** | **hypothetical protein** | **44542** | **44694** |
|  |  | cmeB | CJJ81176_0389 | RND efflux system, inner membrane transporter CmeB | 336608 | 339730 |
|  |  | tuf | CJJ81176_0499 | elongation factor Tu | 436080 | 437279 |
|  |  | sucC | CJJ81176_0558 | succinyl-CoA synthetase subunit beta | 494538 | 495701 |
|  |  | sucD | CJJ81176_0559 | succinyl-CoA synthase, alpha subunit | 495711 | 496580 |
|  |  | ftsZ | CJJ81176_0719 | cell division protein FtsZ | 649668 | 650780 |
|  |  | CJJ81176_0720 | CJJ81176_0720 | flagellar basal-body rod protein | 650936 | 651748 |
|  |  | **CJJ81176_0764** | **CJJ81176_0764** | **putative outer-membrane protein** | **690715** | **692241** |
|  |  | CJJ81176_0992 | CJJ81176_0992 | putative periplasmic protein | 911595 | 912038 |
|  |  | CJJ81176_1741 | CJJ81176_1741 | putative periplasmic protein | 912229 | 913098 |
|  |  | CJJ81176_1743 | CJJ81176_1743 | putative periplasmic protein | 913709 | 915370 |
|  |  | CJJ81176_1744 | CJJ81176_1744 | hypothetical protein | 915632 | 917287 |
|  |  | CJJ81176_1171 | CJJ81176_1171 | cytochrome oxidase maturation protein, cbb3-type, putative | 1087378 | 1087584 |
|  |  | CJJ81176_1172 | CJJ81176_1172 | heavy metal translocating P-type ATPase | 1087581 | 1089932 |
|  |  | CJJ81176_1288 | CJJ81176_1288 | RelA/SpoT family protein | 1206254 | 1208425 |
|  |  | pyrH | CJJ81176_1290 | uridylate kinase | 1208668 | 1209387 |
|  |  | dxr | CJJ81176_1345 | 1-deoxy-D-xylulose 5-phosphate reductoisomerase | 1263017 | 1264087 |
|  |  | CJJ81176_1347 | CJJ81176_1347 | hypothetical protein | 1264818 | 1265156 |
|  |  | CJJ81176_1458 | CJJ81176_1458 | hypothetical protein | 1378256 | 1378690 |
|  |  | CJJ81176_1459 | CJJ81176_1459 | flagellar hook-associated protein FlgK | 1378700 | 1380526 |
|  |  | CJJ81176_1534 | CJJ81176_1534 | type I restriction-modification enzyme, R subunit | 1454125 | 1457220 |
|  | GCAAGG | **rrlA** | **CJJ81176_1707** | **23S ribosomal RNA** | **41513** | **44306** |
|  |  | cmeB | CJJ81176_0389 | RND efflux system, inner membrane transporter CmeB | 336608 | 339730 |
|  |  | CJJ81176_0720 | CJJ81176_0720 | flagellar basal-body rod protein | 650936 | 651748 |
|  |  | CJJ81176_1172 | CJJ81176_1172 | heavy metal translocating P-type ATPase | 1087581 | 1089932 |
|  |  | dxr | CJJ81176_1345 | 1-deoxy-D-xylulose 5-phosphate reductoisomerase | 1263017 | 1264087 |
|  |  | CJJ81176_1534 | CJJ81176_1534 | type I restriction-modification enzyme, R subunit | 1454125 | 1457220 |
|  | AGTNNNNNNRTTG | CJJ81176_0992 | CJJ81176_0992 | putative periplasmic protein | 911595 | 912038 |
|  |  | CJJ81176_1743 | CJJ81176_1743 | putative periplasmic protein | 913709 | 915370 |
|  |  | CJJ81176_1288 | CJJ81176_1288 | RelA/SpoT family protein | 1206254 | 1208425 |
|  | CAAYNNNNNNACT | **CJJ81176_0764** | **CJJ81176_0764** | **putative outer-membrane protein** | **690715** | **692241** |
|  | GGRCA | **rrlA** | **CJJ81176_1707** | **23S ribosomal RNA** | **41513** | **44306** |
|  |  | cmeB | CJJ81176_0389 | RND efflux system, inner membrane transporter CmeB | 336608 | 339730 |
|  |  | tuf | CJJ81176_0499 | elongation factor Tu | 436080 | 437279 |
|  |  | sucD | CJJ81176_0559 | succinyl-CoA synthase, alpha subunit | 495711 | 496580 |
|  |  | **rrsB** | **CJJ81176_1711** | **16S ribosomal RNA** | **395765** | **397275** |
|  |  | **CJJ81176_0764** | **CJJ81176_0764** | **putative outer-membrane protein** | **690715** | **692241** |
|  |  | **rrsC** | **CJJ81176_1724** | **16S ribosomal RNA** | **692913** | **694425** |
|  |  | **rrlC** | **CJJ81176_1727** | **23S ribosomal RNA** | **695271** | **698066** |
|  |  | CJJ81176_0993 | CJJ81176_0993 | putative periplasmic protein | 912068 | 912184 |
|  |  | CJJ81176_1743 | CJJ81176_1743 | putative periplasmic protein | 913709 | 915370 |
|  |  | CJJ81176_1288 | CJJ81176_1288 | RelA/SpoT family protein | 1206254 | 1208425 |
|  |  | pyrH | CJJ81176_1290 | uridylate kinase | 1208668 | 1209387 |
|  |  | dxr | CJJ81176_1345 | 1-deoxy-D-xylulose 5-phosphate reductoisomerase | 1263017 | 1264087 |
|  |  | CJJ81176_1459 | CJJ81176_1459 | flagellar hook-associated protein FlgK | 1378700 | 1380526 |
|  |  | CJJ81176_1534 | CJJ81176_1534 | type I restriction-modification enzyme, R subunit | 1454125 | 1457220 |

- 1. **Supplementary Table 7. Hypomethylated genes detected in IA3902, 11168, and 81-176.^*^**
     1. ***Bolded genes** are found in major hypomethylated areas

| Strain | Gene name | Locus tag | Gene description |
| --- | --- | --- | --- |
| IA3902 | CJSA_0145 | CJSA_0145 | tetrapyrrole methylase family protein |
|  | CJSA_0147 | CJSA_0147 | 16S ribosomal RNA methyltransferase RsmE |
|  | purQ | CJSA_0484 | phosphoribosylformylglycinamidine synthase I |
|  | CJSA_0485 | CJSA_0485 | hypothetical protein |
|  | **CJSA_0658** | **CJSA_0658** | **hypothetical protein** |
|  | **ftsA** | **CJSA_0659** | **cell division protein FtsA** |
|  | **flgG2** | **CJSA_0661** | **flagellar basal-body rod protein** |
|  | **glnA** | **CJSA_0663** | **glutamine synthetase, type I** |
|  | **gyrA** | **CJSA_0970** | **DNA gyrase subunit A** |
|  | folC | CJSA_1030 | olylpolyglutamate synthase/dihydrofolate synthase |
|  | CJSA_1031 | CJSA_1031 | hypothetical protein |
|  | CJSA_1223 | CJSA_1223 | hypothetical protein |
|  | upp | CJSA_1224 | uracil phosphoribosyltransferase |
|  | **CJSA_1309** | **CJSA_1309** | **putative multidrug efflux transporter** |
|  | **CJSA_1311** | **CJSA_1311** | **putative ferredoxin** |
|  | **selA** | **CJSA_1312** | **selenocysteine synthase** |
|  | CJSA_1496 | CJSA_1496 | putative peptide ABC-transport system periplasmic peptide-binding protein |
|  | topA | CJSA_1595 | DNA topoisomerase I |
|  | CJSA_1619 | CJSA_1619 | putative metallo-beta-lactamase family protein |
|  | ksgA | CJSA_1620 | dimethyladenosine transferase |
|  | CJSA_1621 | CJSA_1621 | hypothetical protein |
| 11168 | cmeB | Cj0366c | inner membrane efflux transporter CmeB |
|  | frdC | Cj0408 | fumarate reductase cytochrome b-556 subunit |
|  | frdA | Cj0409 | fumarate reductase flavoprotein subunit |
|  | tuf | Cj0470 | elongation factor Tu |
|  | Cj0939c | Cj0939c | hypothetical protein |
|  | glnP | Cj0940c | glutamine transporter permease |
|  | ssb | Cj1071 | single-stranded DNA-binding protein |
|  | rpsR | Cj1072 | 30S ribosomal protein S18 |
|  | lon | Cj1073c | ATP-dependent protease La |
|  | groEL | Cj1221 | molecular chaperone GroEL |
|  | Cj1389 | Cj1389 | pseudo |
|  | metC | Cj1393 | cystathionine beta-lyase |
|  | hsdR | Cj1549c | type I restriction enzyme R protein |
|  | mloB | Cj1552c | hypothetical protein |
|  | hsdM | Cj1553c | type I restriction enzyme M protein |
|  | **Cjr01** | **Cjr01** | **16S ribosomal RNA** |
|  | **Cjr02** | **Cjr02** | **23S ribosomal RNA** |
|  | **Cjr04** | **Cjr04** | **16S ribosomal RNA** |
|  | **Cjr05** | **Cjr05** | **23S ribosomal RNA** |
|  | **Cjr07** | **Cjr07** | **16S ribosomal RNA** |
|  | **Cjr08** | **Cjr08** | **23S ribosomal RNA** |
| 81-176 | **CJJ81176_0061** | **CJJ81176_0061** | **hypothetical protein** |
|  | cmeB | CJJ81176_0389 | RND efflux system, inner membrane transporter CmeB |
|  | tuf | CJJ81176_0499 | elongation factor Tu |
|  | sucC | CJJ81176_0558 | succinyl-CoA synthetase subunit beta |
|  | sucD | CJJ81176_0559 | succinyl-CoA synthase, alpha subunit |
|  | ftsZ | CJJ81176_0719 | cell division protein FtsZ |
|  | CJJ81176_0720 | CJJ81176_0720 | flagellar basal-body rod protein |
|  | **CJJ81176_0764** | **CJJ81176_0764** | **putative outer-membrane protein** |
|  | CJJ81176_0992 | CJJ81176_0992 | putative periplasmic protein |
|  | CJJ81176_0993 | CJJ81176_0993 | putative periplasmic protein |
|  | CJJ81176_1171 | CJJ81176_1171 | cytochrome oxidase maturation protein, cbb3-type, putative |
|  | CJJ81176_1172 | CJJ81176_1172 | heavy metal translocating P-type ATPase |
|  | CJJ81176_1288 | CJJ81176_1288 | RelA/SpoT family protein |
|  | pyrH | CJJ81176_1290 | uridylate kinase |
|  | dxr | CJJ81176_1345 | 1-deoxy-D-xylulose 5-phosphate reductoisomerase |
|  | CJJ81176_1347 | CJJ81176_1347 | hypothetical protein |
|  | CJJ81176_1458 | CJJ81176_1458 | hypothetical protein |
|  | CJJ81176_1459 | CJJ81176_1459 | flagellar hook-associated protein FlgK |
|  | CJJ81176_1534 | CJJ81176_1534 | type I restriction-modification enzyme, R subunit |
|  | **rrlA** | **CJJ81176_1707** | **23S ribosomal RNA** |
|  | **rrsB** | **CJJ81176_1711** | **16S ribosomal RNA** |
|  | **rrsC** | **CJJ81176_1724** | **16S ribosomal RNA** |
|  | **rrlC** | **CJJ81176_1727** | **23S ribosomal RNA** |
|  | CJJ81176_1741 | CJJ81176_1741 | putative periplasmic protein |
|  | CJJ81176_1743 | CJJ81176_1743 | putative periplasmic protein |
|  | CJJ81176_1744 | CJJ81176_1744 | hypothetical protein |

- 1. **Supplementary Table 8. Motifs and associated hypermethylated genes detected in IA3902, 11168, and 81-176.**

| Strain | Motif | Gene name | Locus tag | Gene description | Start Position | End Position |
| --- | --- | --- | --- | --- | --- | --- |
| IA3902 | TAAYNNNNNTGC | dnaA | CJSA_0001 | chromosomal replication initiator protein DnaA | 1 | 1323 |
|  |  | CJSA_0008 | CJSA_0008 | hypothetical protein | 12644 | 14395 |
|  |  | pyrG | CJSA_0027 | CTP synthetase | 34393 | 36024 |
|  |  | CJSA_pVir0016 | CJSA_pVir0016 | hypothetical protein | 12517 | 13053 |
|  | GCANNNNNRTTA | CJSA_0008 | CJSA_0008 | hypothetical protein | 12644 | 14395 |
|  |  | pyrG | CJSA_0027 | CTP synthetase | 34393 | 36024 |
|  |  | CJSA_pVir0035 | CJSA_pVir0035 | hypothetical protein | 25990 | 26298 |
|  | RAATTY | dnaA | CJSA_0001 | chromosomal replication initiator protein DnaA | 1 | 1323 |
|  |  | CJSA_0008 | CJSA_0008 | hypothetical protein | 12644 | 14395 |
|  |  | gltD | CJSA_0009 | glutamate synthase subunit beta | 14398 | 15843 |
|  |  | rnhB | CJSA_0010 | ribonuclease HII | 15844 | 16419 |
|  |  | CJSA_0015 | CJSA_0015 | hypothetical protein | 19867 | 21093 |
|  |  | CJSA_0016 | CJSA_0016 | ExsB protein | 21170 | 21844 |
|  |  | dsbI | CJSA_0017 | DsbB family disulfide bond formation protein | 21865 | 23391 |
|  |  | CJSA_0019 | CJSA_0019 | methyl-accepting chemotaxis protein | 23676 | 25454 |
|  |  | ccpA-1 | CJSA_0020 | cytochrome c551 peroxidase | 25444 | 26358 |
|  |  | thyX | CJSA_0026 | FAD-dependent thymidylate synthase | 33650 | 34273 |
|  |  | pyrG | CJSA_0027 | CTP synthetase | 34393 | 36024 |
|  |  | CJSA_1306 | CJSA_1306 | putative periplasmic toluene tolerance protein | 1313949 | 1314518 |
|  |  | CJSA_1307 | CJSA_1307 | putative integral membrane protein | 1314518 | 1316989 |
|  |  | virB8 | CJSA_pVir0001 | virulence protein | 1 | 678 |
|  |  | virB9 | CJSA_pVir0002 | VirB9 | 678 | 1748 |
|  |  | CJSA_pVir0016 | CJSA_pVir0016 | hypothetical protein | 12517 | 13053 |
|  |  | CJSA_pVir0017 | CJSA_pVir0017 | phage protein | 13056 | 13502 |
|  |  | CJSA_pVir0018 | CJSA_pVir0018 | hypothetical protein | 13499 | 13936 |
|  |  | CJSA_pVir0021 | CJSA_pVir0021 | hypothetical protein | 14973 | 15380 |
|  |  | CJSA_pVir0022 | CJSA_pVir0022 | hypothetical protein | 15391 | 15714 |
|  |  | CJSA_pVir0023 | CJSA_pVir0023 | hypothetical protein | 15783 | 16016 |
|  |  | CJSA_pVir0028 | CJSA_pVir0028 | hypothetical protein | 20904 | 21860 |
|  |  | CJSA_pVir0029 | CJSA_pVir0029 | TrbM-like protein | 21964 | 22746 |
|  |  | CJSA_pVir0032 | CJSA_pVir0032 | hypothetical protein | 24733 | 25161 |
|  |  | CJSA_pVir0033 | CJSA_pVir0033 | hypothetical protein | 25412 | 25537 |
|  |  | CJSA_pVir0034 | CJSA_pVir0034 | hypothetical protein | 25632 | 25976 |
|  |  | CJSA_pVir0035 | CJSA_pVir0035 | hypothetical protein | 25990 | 26298 |
|  |  | virB4 | CJSA_pVir0052 | VirB4 | 34563 | 37031 |
|  | CAAAYG | dnaA | CJSA_0001 | chromosomal replication initiator protein DnaA | 1 | 1323 |
|  |  | gltD | CJSA_0009 | glutamate synthase subunit beta | 14398 | 15843 |
|  |  | CJSA_0015 | CJSA_0015 | hypothetical protein | 19867 | 21093 |
|  |  | CJSA_0016 | CJSA_0016 | ExsB protein | 21170 | 21844 |
|  |  | ccpA-1 | CJSA_0020 | cytochrome c551 peroxidase | 25444 | 26358 |
|  |  | CJSA_1307 | CJSA_1307 | putative integral membrane protein | 1314518 | 1316989 |
|  |  | virB8 | CJSA_pVir0001 | virulence protein | 1 | 678 |
|  |  | virB9 | CJSA_pVir0002 | VirB9 | 678 | 1748 |
|  |  | CJSA_pVir0022 | CJSA_pVir0022 | hypothetical protein | 15391 | 15714 |
|  |  | CJSA_pVir0029 | CJSA_pVir0029 | TrbM-like protein | 21964 | 22746 |
|  |  | CJSA_pVir0033 | CJSA_pVir0033 | hypothetical protein | 25412 | 25537 |
|  |  | CJSA_pVir0035 | CJSA_pVir0035 | hypothetical protein | 25990 | 26298 |
|  |  | CJSA_pVir0050 | CJSA_pVir0050 | hypothetical protein | 34006 | 34311 |
|  |  | virB4 | CJSA_pVir0052 | VirB4 | 34563 | 37031 |
|  | GAGNNNNNRTG | ccpA-1 | CJSA_0020 | cytochrome c551 peroxidase | 25444 | 26358 |
|  |  | virB9 | CJSA_pVir0002 | VirB9 | 678 | 1748 |
|  |  | CJSA_pVir0018 | CJSA_pVir0018 | hypothetical protein | 13499 | 13936 |
|  |  | virB4 | CJSA_pVir0052 | VirB4 | 34563 | 37031 |
|  | CAYNNNNNCTC | rnhB | CJSA_0010 | ribonuclease HII | 15844 | 16419 |
|  |  | dsbI | CJSA_0017 | DsbB family disulfide bond formation protein | 21865 | 23391 |
|  |  | ccpA-1 | CJSA_0020 | cytochrome c551 peroxidase | 25444 | 26358 |
|  |  | thyX | CJSA_0026 | FAD-dependent thymidylate synthase | 33650 | 34273 |
|  |  | pyrG | CJSA_0027 | CTP synthetase | 34393 | 36024 |
|  |  | virB8 | CJSA_pVir0001 | virulence protein | 1 | 678 |
|  | GAAGAA | dnaA | CJSA_0001 | chromosomal replication initiator protein DnaA | 1 | 1323 |
|  |  | CJSA_0008 | CJSA_0008 | hypothetical protein | 12644 | 14395 |
|  |  | gltD | CJSA_0009 | glutamate synthase subunit beta | 14398 | 15843 |
|  |  | CJSA_0016 | CJSA_0016 | ExsB protein | 21170 | 21844 |
|  |  | CJSA_0019 | CJSA_0019 | methyl-accepting chemotaxis protein | 23676 | 25454 |
|  |  | CJSA_1306 | CJSA_1306 | putative periplasmic toluene tolerance protein | 1313949 | 1314518 |
|  |  | virB8 | CJSA_pVir0001 | virulence protein | 1 | 678 |
|  |  | virB9 | CJSA_pVir0002 | VirB9 | 678 | 1748 |
|  |  | CJSA_pVir0021 | CJSA_pVir0021 | hypothetical protein | 14973 | 15380 |
|  |  | CJSA_pVir0022 | CJSA_pVir0022 | hypothetical protein | 15391 | 15714 |
|  |  | CJSA_pVir0028 | CJSA_pVir0028 | hypothetical protein | 20904 | 21860 |
|  |  | CJSA_pVir0032 | CJSA_pVir0032 | hypothetical protein | 24733 | 25161 |
|  |  | CJSA_pVir0034 | CJSA_pVir0034 | hypothetical protein | 25632 | 25976 |
|  |  | CJSA_pVir0050 | CJSA_pVir0050 | hypothetical protein | 34006 | 34311 |
|  |  | CJSA_pVir0051 | CJSA_pVir0051 | hypothetical protein | 34311 | 34553 |
|  |  | virB4 | CJSA_pVir0052 | VirB4 | 34563 | 37031 |
| 11168 | TAAYNNNNNTGC | none |  |  |  |  |
|  | GCANNNNNRTTA | none |  |  |  |  |
|  | RAATTY | pabB | Cj0862c | para-aminobenzoate synthase subunit I | 807497 | 809281 |
|  | GKAAYG | none |  |  |  |  |
|  | GAGNNNNNGT | none |  |  |  |  |
|  | ACNNNNNCTC | pabB | Cj0862c | para-aminobenzoate synthase subunit I | 807497 | 809281 |
|  | GAGANNNNGMT | none |  |  |  |  |
| 81-176 | TAAYNNNNNTGC | none |  |  |  |  |
|  | GCANNNNNRTTA | CJJ81176_0229 | CJJ81176_0229 | recombination factor protein RarA | 199358 | 200539 |
|  |  | CJJ81176_0326 | CJJ81176_0326 | biotin biosynthesis protein BioC | 280947 | 281633 |
|  | RAATTY | miaA | CJJ81176_0201 | tRNA delta(2)-isopentenylpyrophosphate transferase | 174935 | 175804 |
|  |  | miaA | CJJ81176_0202 | hypothetical protein | 175790 | 176353 |
|  |  | CJJ81176_0229 | CJJ81176_0229 | recombination factor protein RarA | 199358 | 200539 |
|  |  | CJJ81176_0230 | CJJ81176_0230 | hypothetical protein | 200539 | 201666 |
|  |  | CJJ81176_0326 | CJJ81176_0326 | biotin biosynthesis protein BioC | 280947 | 281633 |
|  |  | CJJ81176_0327 | CJJ81176_0327 | hypothetical protein | 281630 | 282241 |
|  |  | CJJ81176_1340 | CJJ81176_1340 | motility accessory factor | 1257013 | 1258839 |
|  | GCAAGG | CJJ81176_0229 | CJJ81176_0229 | recombination factor protein RarA | 199358 | 200539 |
|  | AGTNNNNNNRTTG | CJJ81176_1340 | CJJ81176_1340 | motility accessory factor | 1257013 | 1258839 |
|  | CAAYNNNNNNACT | CJJ81176_0230 | CJJ81176_0230 | hypothetical protein | 200539 | 201666 |
|  | GGRCA | CJJ81176_0229 | CJJ81176_0229 | recombination factor protein RarA | 199358 | 200539 |
|  |  | CJJ81176_0230 | CJJ81176_0230 | hypothetical protein | 200539 | 201666 |
|  |  | CJJ81176_0327 | CJJ81176_0327 | hypothetical protein | 281630 | 282241 |
|  |  | CJJ81176_1340 | CJJ81176_1340 | motility accessory factor | 1257013 | 1258839 |

- 1. **Supplementary Table 9. Hypermethylated genes detected in IA3902, 11168, and 81-176.**

| Strain | Gene name | Locus tag | Gene description |
| --- | --- | --- | --- |
| IA3902 | dnaA | CJSA_0001 | chromosomal replication initiator protein DnaA |
|  | CJSA_0008 | CJSA_0008 | hypothetical protein |
|  | gltD | CJSA_0009 | glutamate synthase subunit beta |
|  | rnhB | CJSA_0010 | ribonuclease HII |
|  | CJSA_0015 | CJSA_0015 | hypothetical protein |
|  | CJSA_0016 | CJSA_0016 | ExsB protein |
|  | dsbI | CJSA_0017 | DsbB family disulfide bond formation protein |
|  | CJSA_0019 | CJSA_0019 | methyl-accepting chemotaxis protein |
|  | ccpA-1 | CJSA_0020 | cytochrome c551 peroxidase |
|  | thyX | CJSA_0026 | FAD-dependent thymidylate synthase |
|  | pyrG | CJSA_0027 | CTP synthetase |
|  | CJSA_1306 | CJSA_1306 | putative periplasmic toluene tolerance protein |
|  | CJSA_1307 | CJSA_1307 | putative integral membrane protein |
|  | virB8 | CJSA_pVir0001 | virulence protein |
|  | virB9 | CJSA_pVir0002 | VirB9 |
|  | CJSA_pVir0016 | CJSA_pVir0016 | hypothetical protein |
|  | CJSA_pVir0017 | CJSA_pVir0017 | phage protein |
|  | CJSA_pVir0018 | CJSA_pVir0018 | hypothetical protein |
|  | CJSA_pVir0021 | CJSA_pVir0021 | hypothetical protein |
|  | CJSA_pVir0022 | CJSA_pVir0022 | hypothetical protein |
|  | CJSA_pVir0023 | CJSA_pVir0023 | hypothetical protein |
|  | CJSA_pVir0028 | CJSA_pVir0028 | hypothetical protein |
|  | CJSA_pVir0029 | CJSA_pVir0029 | TrbM-like protein |
|  | CJSA_pVir0032 | CJSA_pVir0032 | hypothetical protein |
|  | CJSA_pVir0033 | CJSA_pVir0033 | hypothetical protein |
|  | CJSA_pVir0034 | CJSA_pVir0034 | hypothetical protein |
|  | CJSA_pVir0035 | CJSA_pVir0035 | hypothetical protein |
|  | CJSA_pVir0050 | CJSA_pVir0050 | hypothetical protein |
|  | CJSA_pVir0051 | CJSA_pVir0051 | hypothetical protein |
|  | virB4 | CJSA_pVir0052 | VirB4 |
| 11168 | pabB | Cj0862c | para-aminobenzoate synthase subunit I |
| 81-176 | CJJ81176_0229 | CJJ81176_0229 | recombination factor protein RarA |
|  | CJJ81176_0326 | CJJ81176_0326 | biotin biosynthesis protein BioC |
|  | miaA | CJJ81176_0201 | tRNA delta(2)-isopentenylpyrophosphate transferase |
|  | miaA | CJJ81176_0202 | hypothetical protein |
|  | CJJ81176_0230 | CJJ81176_0230 | hypothetical protein |
|  | CJJ81176_0327 | CJJ81176_0327 | hypothetical protein |
|  | CJJ81176_1340 | CJJ81176_1340 | motility accessory factor |

- 1. **Supplementary Table 10. Summary of methylated genes from each role category in IA3902 (A), 11168 (B) and 81-176 (C).**
     1. *Motif is homologous with IA3902 motif
     2. **Motif has no homology with other *C. jejuni* strains’ motifs
     3. A.

|  |  | **% Total methylated genes by Role Category** | | | | | | | |
| --- | --- | --- | --- | --- | --- | --- | --- | --- | --- |
|  |  | **IA3902** | | | | | | | |
| **#** | **Role Category** | **All motifs** | **CAYNNNNNCTC** | **GAGNNNNNRTG** | **TAAYNNNNNTGC** | **GCANNNNNRTTA** | **RAATTY** | **CAAAYG** | **GAAGAA** |
| 1 | Amino acid biosynthesis | 100.00 | 19.12 | 16.18 | 16.18 | 13.24 | 100.00 | 38.24 | 47.06 |
| 2 | Biosynthesis of cofactors, prosthetic groups, and carriers | 96.10 | 18.18 | 24.68 | 11.69 | 6.49 | 93.51 | 37.66 | 45.45 |
| 3 | Cell envelope | 97.67 | 14.88 | 14.42 | 15.81 | 13.02 | 95.35 | 35.81 | 38.60 |
| 4 | Cellular processes | 98.28 | 27.59 | 17.24 | 10.34 | 17.24 | 96.55 | 31.03 | 41.38 |
| 5 | Central intermediary metabolism | 100.00 | 16.67 | 33.33 | 11.11 | 16.67 | 100.00 | 38.89 | 61.11 |
| 6 | DNA metabolism, replication, and repair | 100.00 | 20.00 | 20.00 | 9.09 | 20.00 | 100.00 | 45.45 | 60.00 |
| 7 | Energy metabolism | 99.04 | 20.19 | 12.50 | 8.65 | 17.31 | 97.12 | 34.62 | 43.27 |
| 8 | Fatty acid biosynthesis | 100.00 | 17.24 | 17.24 | 20.69 | 17.24 | 100.00 | 41.38 | 20.69 |
| 9 | Hypothetical proteins | 96.80 | 11.20 | 9.60 | 8.80 | 10.40 | 95.20 | 26.40 | 26.40 |
| 10 | Mobile and extrachromosomal element functions | 0.00 | 0.00 | 0.00 | 0.00 | 0.00 | 0.00 | 0.00 | 0.00 |
| 11 | Protein fate | 100.00 | 17.39 | 21.74 | 10.87 | 15.22 | 100.00 | 34.78 | 50.00 |
| 12 | Purines, pyrimidines, nucleosides, and nucleotides | 97.62 | 14.29 | 21.43 | 7.14 | 26.19 | 97.62 | 33.33 | 47.62 |
| 13 | Regulatory functions | 100.00 | 11.54 | 7.69 | 15.38 | 11.54 | 100.00 | 46.15 | 50.00 |
| 14 | Transcription and RNA metabolism | 100.00 | 41.18 | 23.53 | 23.53 | 29.41 | 94.12 | 41.18 | 41.18 |
| 15 | Transport and binding proteins | 100.00 | 16.55 | 21.38 | 13.79 | 11.03 | 100.00 | 47.59 | 44.83 |
| 16 | General function | 99.44 | 21.47 | 24.29 | 12.43 | 15.25 | 97.74 | 34.46 | 45.76 |
| 17 | Conserved hypothetical proteins | 96.27 | 17.91 | 11.19 | 7.46 | 8.96 | 94.78 | 22.39 | 23.88 |
| 18 | rRNA and stable RNAs | 0.00 | 0.00 | 0.00 | 0.00 | 0.00 | 0.00 | 0.00 | 0.00 |
| 19 | Protein synthesis | 96.12 | 10.08 | 15.50 | 17.83 | 6.98 | 95.35 | 38.76 | 35.66 |
| 20 | Signal transduction | 100.00 | 18.75 | 12.50 | 25.00 | 12.50 | 100.00 | 50.00 | 37.50 |
| 21 | Chemotaxis and motility | 98.81 | 11.90 | 22.62 | 11.90 | 16.67 | 95.24 | 36.90 | 53.57 |
| 22 | Small molecule degradation | 100.00 | 50.00 | 0.00 | 0.00 | 33.33 | 100.00 | 50.00 | 33.33 |
| 24 | Pseudogenes/degenerate CDS | 100.00 | 7.14 | 7.14 | 14.29 | 14.29 | 100.00 | 57.14 | 28.57 |

- - 1. B.

|  |  | **% Total methylated genes by Role Category** | | | | | | | |
| --- | --- | --- | --- | --- | --- | --- | --- | --- | --- |
|  |  | **11168** | | | | | | | |
| **#** | **Role Category** | **All motifs** | **ACNNNNNCTC*** | **GAGNNNNNGT*** | **TAAYNNNNNTGC*** | **GCANNNNNRTTA*** | **RAATTY*** | **GKAAYG*** | **GAGANNNNGMT**** |
| 1 | Amino acid biosynthesis | 98.51 | 19.40 | 40.30 | 17.91 | 7.46 | 97.01 | 44.78 | 7.46 |
| 2 | Biosynthesis of cofactors, prosthetic groups, and carriers | 100.00 | 6.76 | 27.03 | 9.46 | 5.41 | 100.00 | 27.03 | 8.11 |
| 3 | Cell envelope | 98.19 | 19.46 | 28.05 | 14.03 | 15.38 | 97.74 | 34.39 | 7.69 |
| 4 | Cellular processes | 100.00 | 16.36 | 20.00 | 27.27 | 1.82 | 100.00 | 38.18 | 5.45 |
| 5 | Central intermediary metabolism | 100.00 | 18.75 | 43.75 | 18.75 | 6.25 | 100.00 | 68.75 | 0.00 |
| 6 | DNA metabolism, replication, and repair | 100.00 | 27.78 | 35.19 | 20.37 | 18.52 | 100.00 | 40.74 | 7.41 |
| 7 | Energy metabolism | 99.05 | 19.05 | 41.90 | 17.14 | 7.62 | 97.14 | 52.38 | 10.48 |
| 8 | Fatty acid biosynthesis | 100.00 | 10.71 | 25.00 | 17.86 | 7.14 | 96.43 | 42.86 | 3.57 |
| 9 | Hypothetical proteins | 96.49 | 14.04 | 13.16 | 9.65 | 7.89 | 95.61 | 29.82 | 1.75 |
| 10 | Mobile and extrachromosomal element functions | 0.00 | 0.00 | 0.00 | 0.00 | 0.00 | 0.00 | 0.00 | 0.00 |
| 11 | Protein fate | 97.78 | 15.56 | 42.22 | 8.89 | 4.44 | 97.78 | 28.89 | 8.89 |
| 12 | Purines, pyrimidines, nucleosides, and nucleotides | 100.00 | 30.95 | 38.10 | 14.29 | 14.29 | 100.00 | 54.76 | 7.14 |
| 13 | Regulatory functions | 100.00 | 12.00 | 16.00 | 4.00 | 4.00 | 100.00 | 20.00 | 0.00 |
| 14 | Transcription and RNA metabolism | 100.00 | 29.41 | 41.18 | 11.76 | 23.53 | 94.12 | 35.29 | 0.00 |
| 15 | Transport and binding proteins | 100.00 | 23.91 | 36.96 | 13.04 | 12.32 | 99.28 | 43.48 | 5.07 |
| 16 | General function | 100.00 | 14.79 | 28.99 | 14.79 | 9.47 | 98.82 | 31.95 | 10.06 |
| 17 | Conserved hypothetical proteins | 94.78 | 15.67 | 19.40 | 12.69 | 8.21 | 91.79 | 22.39 | 5.22 |
| 18 | rRNA and stable RNAs | 0.00 | 0.00 | 0.00 | 0.00 | 0.00 | 0.00 | 0.00 | 0.00 |
| 19 | Protein synthesis | 94.57 | 19.38 | 27.13 | 6.98 | 7.75 | 92.25 | 37.21 | 8.53 |
| 20 | Signal transduction | 100.00 | 6.25 | 37.50 | 25.00 | 25.00 | 100.00 | 25.00 | 0.00 |
| 21 | Chemotaxis and motility | 97.62 | 40.48 | 35.71 | 21.43 | 10.71 | 96.43 | 44.05 | 13.10 |
| 22 | Small molecule degradation | 100.00 | 16.67 | 66.67 | 33.33 | 16.67 | 100.00 | 100.00 | 0.00 |
| 24 | Pseudogenes/degenerate CDS | 100.00 | 17.65 | 35.29 | 17.65 | 17.65 | 100.00 | 64.71 | 11.76 |

- - 1. C.

|  |  | **% Total methylated genes by Role Category** | | | | | | | |
| --- | --- | --- | --- | --- | --- | --- | --- | --- | --- |
|  |  | **81-176** | | | | | | | |
| **#** | **Role Category** | **All motifs** | **CAAYNNNNNNACT**** | **AGTNNNNNNRTTG**** | **TAAYNNNNNTGC*** | **GCANNNNNRTTA*** | **RAATTY*** | **GCAAGG*** | **GGRCA*** |
| 1 | Amino acid biosynthesis | 98.51 | 8.96 | 10.45 | 14.93 | 10.45 | 98.51 | 28.36 | 52.24 |
| 2 | Biosynthesis of cofactors, prosthetic groups, and carriers | 100.00 | 5.19 | 9.09 | 10.39 | 5.19 | 100.00 | 16.88 | 51.95 |
| 3 | Cell envelope | 98.43 | 10.99 | 10.99 | 12.57 | 14.14 | 98.43 | 19.37 | 43.98 |
| 4 | Cellular processes | 100.00 | 7.69 | 5.77 | 28.85 | 7.69 | 100.00 | 15.38 | 36.54 |
| 5 | Central intermediary metabolism | 100.00 | 12.50 | 6.25 | 25.00 | 6.25 | 100.00 | 18.75 | 75.00 |
| 6 | DNA metabolism, replication, and repair | 100.00 | 9.62 | 13.46 | 17.31 | 13.46 | 100.00 | 28.85 | 61.54 |
| 7 | Energy metabolism | 99.03 | 9.71 | 9.71 | 17.48 | 5.83 | 98.06 | 26.21 | 56.31 |
| 8 | Fatty acid biosynthesis | 96.55 | 0.00 | 6.90 | 20.69 | 6.90 | 96.55 | 3.45 | 48.28 |
| 9 | Hypothetical proteins | 98.88 | 5.62 | 3.37 | 13.48 | 10.11 | 95.51 | 11.24 | 33.71 |
| 10 | Mobile and extrachromosomal element functions | 0.00 | 0.00 | 0.00 | 0.00 | 0.00 | 0.00 | 0.00 | 0.00 |
| 11 | Protein fate | 97.78 | 6.67 | 22.22 | 11.11 | 6.67 | 97.78 | 28.89 | 62.22 |
| 12 | Purines, pyrimidines, nucleosides, and nucleotides | 100.00 | 5.00 | 15.00 | 22.50 | 12.50 | 100.00 | 22.50 | 57.50 |
| 13 | Regulatory functions | 100.00 | 9.09 | 22.73 | 9.09 | 4.55 | 100.00 | 22.73 | 50.00 |
| 14 | Transcription and RNA metabolism | 94.12 | 11.76 | 17.65 | 11.76 | 11.76 | 94.12 | 17.65 | 64.71 |
| 15 | Transport and binding proteins | 100.00 | 8.46 | 10.77 | 16.92 | 12.31 | 100.00 | 24.62 | 57.69 |
| 16 | General function | 99.38 | 5.56 | 9.88 | 11.73 | 11.11 | 98.77 | 20.37 | 56.79 |
| 17 | Conserved hypothetical proteins | 98.32 | 8.40 | 7.56 | 15.13 | 9.24 | 94.12 | 26.89 | 33.61 |
| 18 | rRNA and stable RNAs | 88.24 | 11.76 | 11.76 | 5.88 | 11.76 | 82.35 | 23.53 | 58.82 |
| 19 | Protein synthesis | 93.02 | 5.43 | 11.63 | 9.30 | 7.75 | 93.02 | 17.05 | 49.61 |
| 20 | Signal transduction | 100.00 | 6.25 | 12.50 | 25.00 | 18.75 | 100.00 | 31.25 | 25.00 |
| 21 | Chemotaxis and motility | 97.33 | 12.00 | 14.67 | 21.33 | 16.00 | 96.00 | 17.33 | 54.67 |
| 22 | Small molecule degradation | 100.00 | 0.00 | 40.00 | 40.00 | 0.00 | 100.00 | 20.00 | 60.00 |
| 24 | Pseudogenes/degenerate CDS | 0.00 | 0.00 | 0.00 | 0.00 | 0.00 | 0.00 | 0.00 | 0.00 |

- 1. **Supplementary Table 11. Comparisons of gene role categories* between strains IA3902, 11168 and 81-176 by all motifs (A), and by IA3902 motifs TAAYNNNNNTGC (B), GCANNNNNRTTA (C), RAATTY (D), CAAAYG (E), GAGNNNNNRTG (F), CAYNNNNNCTC (G), and GAAGAA (H).**
     1. *Order of role categories per motif are listed from highest to lowest percent methylated genes. Percentages bolded in the top columns are role categories containing the highest percent methylated genes. Percentages bolded in the bottom columns indicate no genes methylated in specific role categories. Motifs of 11168 (GAGANNNNGMT) and 81-176 (CAAYNNNNNNACT and AGTNNNNNNRTTG), did not have any homologues with IA3902 nor were they homologous with one another.
     2. A.

| IA3902 (All motifs) | | 11168 (All motifs) | | 81-176 (All motifs) | |
| --- | --- | --- | --- | --- | --- |
| Role category | Percent methylated genes per category | Role category | Percent methylated genes per category | Role category | Percent methylated genes per category |
| **Amino acid biosynthesis** | **100** | **Biosynthesis of cofactors, prosthetic groups, and carriers** | **100** | **Biosynthesis of cofactors, prosthetic groups, and carriers** | **100** |
| **Central intermediary metabolism** | **100** | **Cellular processes** | **100** | **Cellular processes** | **100** |
| **DNA metabolism, replication, and repair** | **100** | **Central intermediary metabolism** | **100** | **Central intermediary metabolism** | **100** |
| **Fatty acid biosynthesis** | **100** | **DNA metabolism, replication, and repair** | **100** | **DNA metabolism, replication, and repair** | **100** |
| **Protein fate** | **100** | **Fatty acid biosynthesis** | **100** | **Purines, pyrimidines, nucleosides, and nucleotides** | **100** |
| **Pseudogenes/degenerate CDS** | **100** | **General function** | **100** | **Regulatory functions** | **100** |
| **Regulatory functions** | **100** | **Pseudogenes/degenerate CDS** | **100** | **Signal transduction** | **100** |
| **Signal transduction** | **100** | **Purines, pyrimidines, nucleosides, and nucleotides** | **100** | **Small molecule degradation** | **100** |
| **Small molecule degradation** | **100** | **Regulatory functions** | **100** | **Transport and binding proteins** | **100** |
| **Transcription and RNA metabolism** | **100** | **Signal transduction** | **100** | General function | 99.38 |
| **Transport and binding proteins** | **100** | **Small molecule degradation** | **100** | Energy metabolism | 99.03 |
| General function | 99.44 | **Transcription and RNA metabolism** | **100** | Hypothetical proteins | 98.88 |
| Energy metabolism | 99.04 | **Transport and binding proteins** | **100** | Amino acid biosynthesis | 98.51 |
| Chemotaxis and motility | 98.81 | Energy metabolism | 99.05 | Cell envelope | 98.43 |
| Cellular processes | 98.28 | Amino acid biosynthesis | 98.51 | Conserved hypothetical proteins | 98.32 |
| Cell envelope | 97.67 | Cell envelope | 98.19 | Protein fate | 97.78 |
| Purines, pyrimidines, nucleosides, and nucleotides | 97.62 | Protein fate | 97.78 | Chemotaxis and motility | 97.33 |
| Hypothetical proteins | 96.8 | Chemotaxis and motility | 97.62 | Fatty acid biosynthesis | 96.55 |
| Conserved hypothetical proteins | 96.27 | Hypothetical proteins | 96.49 | Transcription and RNA metabolism | 94.12 |
| Protein synthesis | 96.12 | Conserved hypothetical proteins | 94.78 | Protein synthesis | 93.02 |
| Biosynthesis of cofactors, prosthetic groups, and carriers | 96.1 | Protein synthesis | 94.57 | rRNA and stable RNAs | 88.24 |
| **Mobile and extrachromosomal element functions** | **0** | **Mobile and extrachromosomal element functions** | **0** | **Mobile and extrachromosomal element functions** | **0** |
| **rRNA and stable RNAs** | **0** | **rRNA and stable RNAs** | **0** | **Pseudogenes/degenerate CDS** | **0** |

- - 1. B.

| IA3902 (TAAYNNNNNTGC) | | 11168 (TAAYNNNNNTGC) | | 81-176 (TAAYNNNNNTGC) | |
| --- | --- | --- | --- | --- | --- |
| Role category | Percent methylated genes per category | Role category | Percent methylated genes per category | Role category | Percent methylated genes per category |
| **Signal transduction** | **25.00** | **Small molecule degradation** | **33.33** | **Small molecule degradation** | **40.00** |
| Transcription and RNA metabolism | 23.53 | Cellular processes | 27.27 | Cellular processes | 28.85 |
| Fatty acid biosynthesis | 20.69 | Signal transduction | 25.00 | Central intermediary metabolism | 25.00 |
| Protein synthesis | 17.83 | Chemotaxis and motility | 21.43 | Signal transduction | 25.00 |
| Amino acid biosynthesis | 16.18 | DNA metabolism, replication, and repair | 20.37 | Purines, pyrimidines, nucleosides, and nucleotides | 22.50 |
| Cell envelope | 15.81 | Central intermediary metabolism | 18.75 | Chemotaxis and motility | 21.33 |
| Regulatory functions | 15.38 | Amino acid biosynthesis | 17.91 | Fatty acid biosynthesis | 20.69 |
| Pseudogenes/degenerate CDS | 14.29 | Fatty acid biosynthesis | 17.86 | Energy metabolism | 17.48 |
| Transport and binding proteins | 13.79 | Pseudogenes/degenerate CDS | 17.65 | DNA metabolism, replication, and repair | 17.31 |
| General function | 12.43 | Energy metabolism | 17.14 | Transport and binding proteins | 16.92 |
| Chemotaxis and motility | 11.90 | General function | 14.79 | Conserved hypothetical proteins | 15.13 |
| Biosynthesis of cofactors, prosthetic groups, and carriers | 11.69 | Purines, pyrimidines, nucleosides, and nucleotides | 14.29 | Amino acid biosynthesis | 14.93 |
| Central intermediary metabolism | 11.11 | Cell envelope | 14.03 | Hypothetical proteins | 13.48 |
| Protein fate | 10.87 | Transport and binding proteins | 13.04 | Cell envelope | 12.57 |
| Cellular processes | 10.34 | Conserved hypothetical proteins | 12.69 | Transcription and RNA metabolism | 11.76 |
| DNA metabolism, replication, and repair | 9.09 | Transcription and RNA metabolism | 11.76 | General function | 11.73 |
| Hypothetical proteins | 8.80 | Hypothetical proteins | 9.65 | Protein fate | 11.11 |
| Energy metabolism | 8.65 | Biosynthesis of cofactors, prosthetic groups, and carriers | 9.46 | Biosynthesis of cofactors, prosthetic groups, and carriers | 10.39 |
| Conserved hypothetical proteins | 7.46 | Protein fate | 8.89 | Protein synthesis | 9.30 |
| Purines, pyrimidines, nucleosides, and nucleotides | 7.14 | Protein synthesis | 6.98 | Regulatory functions | 9.09 |
| **Small molecule degradation** | **0.00** | Regulatory functions | 4.00 | rRNA and stable RNAs | 5.88 |
| **Mobile and extrachromosomal element functions** | **0.00** | **Mobile and extrachromosomal element functions** | **0.00** | **Mobile and extrachromosomal element functions** | **0.00** |
| **rRNA and stable RNAs** | **0.00** | **rRNA and stable RNAs** | **0.00** | **Pseudogenes/degenerate CDS** | **0.00** |

- - 1. C.

| IA3902 (GCANNNNNRTTA) | | 11168 (GCANNNNNRTTA) | | 81-176 (GCANNNNNRTTA) | |
| --- | --- | --- | --- | --- | --- |
| Role category | Percent methylated genes per category | Role category | Percent methylated genes per category | Role category | Percent methylated genes per category |
| **Small molecule degradation** | **33.33** | **Signal transduction** | **25.00** | **Signal transduction** | **18.75** |
| Transcription and RNA metabolism | 29.41 | Transcription and RNA metabolism | 23.53 | Chemotaxis and motility | 16.00 |
| Purines, pyrimidines, nucleosides, and nucleotides | 26.19 | DNA metabolism, replication, and repair | 18.52 | Cell envelope | 14.14 |
| DNA metabolism, replication, and repair | 20 | Pseudogenes/degenerate CDS | 17.65 | DNA metabolism, replication, and repair | 13.46 |
| Energy metabolism | 17.31 | Small molecule degradation | 16.67 | Purines, pyrimidines, nucleosides, and nucleotides | 12.50 |
| Cellular processes | 17.24 | Cell envelope | 15.38 | Transport and binding proteins | 12.31 |
| Fatty acid biosynthesis | 17.24 | Purines, pyrimidines, nucleosides, and nucleotides | 14.29 | Transcription and RNA metabolism | 11.76 |
| Central intermediary metabolism | 16.67 | Transport and binding proteins | 12.32 | rRNA and stable RNAs | 11.76 |
| Chemotaxis and motility | 16.67 | Chemotaxis and motility | 10.71 | General function | 11.11 |
| General function | 15.25 | General function | 9.47 | Amino acid biosynthesis | 10.45 |
| Protein fate | 15.22 | Conserved hypothetical proteins | 8.21 | Hypothetical proteins | 10.11 |
| Pseudogenes/degenerate CDS | 14.29 | Hypothetical proteins | 7.89 | Conserved hypothetical proteins | 9.24 |
| Amino acid biosynthesis | 13.24 | Protein synthesis | 7.75 | Protein synthesis | 7.75 |
| Cell envelope | 13.02 | Energy metabolism | 7.62 | Cellular processes | 7.69 |
| Signal transduction | 12.5 | Amino acid biosynthesis | 7.46 | Fatty acid biosynthesis | 6.90 |
| Regulatory functions | 11.54 | Fatty acid biosynthesis | 7.14 | Protein fate | 6.67 |
| Transport and binding proteins | 11.03 | Central intermediary metabolism | 6.25 | Central intermediary metabolism | 6.25 |
| Hypothetical proteins | 10.4 | Biosynthesis of cofactors, prosthetic groups, and carriers | 5.41 | Energy metabolism | 5.83 |
| Conserved hypothetical proteins | 8.96 | Protein fate | 4.44 | Biosynthesis of cofactors, prosthetic groups, and carriers | 5.19 |
| Protein synthesis | 6.98 | Regulatory functions | 4.00 | Regulatory functions | 4.55 |
| Biosynthesis of cofactors, prosthetic groups, and carriers | 6.49 | Cellular processes | 1.82 | **Small molecule degradation** | **0.00** |
| **Mobile and extrachromosomal element functions** | **0** | **Mobile and extrachromosomal element functions** | **0.00** | **Mobile and extrachromosomal element functions** | **0.00** |
| **rRNA and stable RNAs** | **0** | **rRNA and stable RNAs** | **0.00** | **Pseudogenes/degenerate CDS** | **0.00** |

- - 1. D.

| IA3902 (RAATTY) | | 11168 (RAATTY) | | 81-176 (RAATTY) | |
| --- | --- | --- | --- | --- | --- |
| Role category | Percent methylated genes per category | Role category | Percent methylated genes per category | Role category | Percent methylated genes per category |
| **Amino acid biosynthesis** | **100.00** | **Biosynthesis of cofactors, prosthetic groups, and carriers** | **100.00** | **Biosynthesis of cofactors, prosthetic groups, and carriers** | **100.00** |
| **Central intermediary metabolism** | **100.00** | **Cellular processes** | **100.00** | **Cellular processes** | **100.00** |
| **DNA metabolism, replication, and repair** | **100.00** | **Central intermediary metabolism** | **100.00** | **Central intermediary metabolism** | **100.00** |
| **Fatty acid biosynthesis** | **100.00** | **DNA metabolism, replication, and repair** | **100.00** | **DNA metabolism, replication, and repair** | **100.00** |
| **Protein fate** | **100.00** | **Pseudogenes/degenerate CDS** | **100.00** | **Purines, pyrimidines, nucleosides, and nucleotides** | **100.00** |
| **Pseudogenes/degenerate CDS** | **100.00** | **Purines, pyrimidines, nucleosides, and nucleotides** | **100.00** | **Regulatory functions** | **100.00** |
| **Regulatory functions** | **100.00** | **Regulatory functions** | **100.00** | **Signal transduction** | **100.00** |
| **Signal transduction** | **100.00** | **Signal transduction** | **100.00** | **Small molecule degradation** | **100.00** |
| **Small molecule degradation** | **100.00** | **Small molecule degradation** | **100.00** | **Transport and binding proteins** | **100.00** |
| **Transport and binding proteins** | **100.00** | Transport and binding proteins | 99.28 | General function | 98.77 |
| General function | 97.74 | General function | 98.82 | Amino acid biosynthesis | 98.51 |
| Purines, pyrimidines, nucleosides, and nucleotides | 97.62 | Protein fate | 97.78 | Cell envelope | 98.43 |
| Energy metabolism | 97.12 | Cell envelope | 97.74 | Energy metabolism | 98.06 |
| Cellular processes | 96.55 | Energy metabolism | 97.14 | Protein fate | 97.78 |
| Cell envelope | 95.35 | Amino acid biosynthesis | 97.01 | Fatty acid biosynthesis | 96.55 |
| Protein synthesis | 95.35 | Fatty acid biosynthesis | 96.43 | Chemotaxis and motility | 96.00 |
| Chemotaxis and motility | 95.24 | Chemotaxis and motility | 96.43 | Hypothetical proteins | 95.51 |
| Hypothetical proteins | 95.20 | Hypothetical proteins | 95.61 | Transcription and RNA metabolism | 94.12 |
| Conserved hypothetical proteins | 94.78 | Transcription and RNA metabolism | 94.12 | Conserved hypothetical proteins | 94.12 |
| Transcription and RNA metabolism | 94.12 | Protein synthesis | 92.25 | Protein synthesis | 93.02 |
| Biosynthesis of cofactors, prosthetic groups, and carriers | 93.51 | Conserved hypothetical proteins | 91.79 | rRNA and stable RNAs | 82.35 |
| **Mobile and extrachromosomal element functions** | **0.00** | **Mobile and extrachromosomal element functions** | **0.00** | **Mobile and extrachromosomal element functions** | **0.00** |
| **rRNA and stable RNAs** | **0.00** | **rRNA and stable RNAs** | **0.00** | **Pseudogenes/degenerate CDS** | **0.00** |

- - 1. E.

| IA3902 (CAAAYG) | | 11168 (GKAAYG) | | 81-176 (GCAAGG) | |
| --- | --- | --- | --- | --- | --- |
| Role category | Percent methylated genes per category | Role category | Percent methylated genes per category | Role category | Percent methylated genes per category |
| **Pseudogenes/degenerate CDS** | **57.14** | **Small molecule degradation** | **100.00** | **Signal transduction** | **31.25** |
| Signal transduction | 50.00 | Central intermediary metabolism | 68.75 | Protein fate | 28.89 |
| Small molecule degradation | 50.00 | Pseudogenes/degenerate CDS | 64.71 | DNA metabolism, replication, and repair | 28.85 |
| Transport and binding proteins | 47.59 | Purines, pyrimidines, nucleosides, and nucleotides | 54.76 | Amino acid biosynthesis | 28.36 |
| Regulatory functions | 46.15 | Energy metabolism | 52.38 | Conserved hypothetical proteins | 26.89 |
| DNA metabolism, replication, and repair | 45.45 | Amino acid biosynthesis | 44.78 | Energy metabolism | 26.21 |
| Fatty acid biosynthesis | 41.38 | Chemotaxis and motility | 44.05 | Transport and binding proteins | 24.62 |
| Transcription and RNA metabolism | 41.18 | Transport and binding proteins | 43.48 | rRNA and stable RNAs | 23.53 |
| Central intermediary metabolism | 38.89 | Fatty acid biosynthesis | 42.86 | Regulatory functions | 22.73 |
| Protein synthesis | 38.76 | DNA metabolism, replication, and repair | 40.74 | Purines, pyrimidines, nucleosides, and nucleotides | 22.50 |
| Amino acid biosynthesis | 38.24 | Cellular processes | 38.18 | General function | 20.37 |
| Biosynthesis of cofactors, prosthetic groups, and carriers | 37.66 | Protein synthesis | 37.21 | Small molecule degradation | 20.00 |
| Chemotaxis and motility | 36.90 | Transcription and RNA metabolism | 35.29 | Cell envelope | 19.37 |
| Cell envelope | 35.81 | Cell envelope | 34.39 | Central intermediary metabolism | 18.75 |
| Protein fate | 34.78 | General function | 31.95 | Transcription and RNA metabolism | 17.65 |
| Energy metabolism | 34.62 | Hypothetical proteins | 29.82 | Chemotaxis and motility | 17.33 |
| General function | 34.46 | Protein fate | 28.89 | Protein synthesis | 17.05 |
| Purines, pyrimidines, nucleosides, and nucleotides | 33.33 | Biosynthesis of cofactors, prosthetic groups, and carriers | 27.03 | Biosynthesis of cofactors, prosthetic groups, and carriers | 16.88 |
| Cellular processes | 31.03 | Signal transduction | 25.00 | Cellular processes | 15.38 |
| Hypothetical proteins | 26.40 | Conserved hypothetical proteins | 22.39 | Hypothetical proteins | 11.24 |
| Conserved hypothetical proteins | 22.39 | Regulatory functions | 20.00 | Fatty acid biosynthesis | 3.45 |
| **Mobile and extrachromosomal element functions** | **0.00** | **Mobile and extrachromosomal element functions** | **0.00** | **Mobile and extrachromosomal element functions** | **0.00** |
| **rRNA and stable RNAs** | **0.00** | **rRNA and stable RNAs** | **0.00** | **Pseudogenes/degenerate CDS** | **0.00** |

- - 1. F.

| IA3902 (GAGNNNNNRTG) | | 11168 (GAGNNNNNGT) | |
| --- | --- | --- | --- |
| Role category | Percent methylated genes per category | Role category | Percent methylated genes per category |
| **Central intermediary metabolism** | **33.33** | **Small molecule degradation** | **66.67** |
| Biosynthesis of cofactors, prosthetic groups, and carriers | 24.68 | Central intermediary metabolism | 43.75 |
| General function | 24.29 | Protein fate | 42.22 |
| Transcription and RNA metabolism | 23.53 | Energy metabolism | 41.90 |
| Chemotaxis and motility | 22.62 | Transcription and RNA metabolism | 41.18 |
| Protein fate | 21.74 | Amino acid biosynthesis | 40.30 |
| Purines, pyrimidines, nucleosides, and nucleotides | 21.43 | Purines, pyrimidines, nucleosides, and nucleotides | 38.10 |
| Transport and binding proteins | 21.38 | Signal transduction | 37.50 |
| DNA metabolism, replication, and repair | 20.00 | Transport and binding proteins | 36.96 |
| Cellular processes | 17.24 | Chemotaxis and motility | 35.71 |
| Fatty acid biosynthesis | 17.24 | Pseudogenes/degenerate CDS | 35.29 |
| Amino acid biosynthesis | 16.18 | DNA metabolism, replication, and repair | 35.19 |
| Protein synthesis | 15.50 | General function | 28.99 |
| Cell envelope | 14.42 | Cell envelope | 28.05 |
| Energy metabolism | 12.50 | Protein synthesis | 27.13 |
| Signal transduction | 12.50 | Biosynthesis of cofactors, prosthetic groups, and carriers | 27.03 |
| Conserved hypothetical proteins | 11.19 | Fatty acid biosynthesis | 25.00 |
| Hypothetical proteins | 9.60 | Cellular processes | 20.00 |
| Regulatory functions | 7.69 | Conserved hypothetical proteins | 19.40 |
| Pseudogenes/degenerate CDS | 7.14 | Regulatory functions | 16.00 |
| **Small molecule degradation** | **0** | Hypothetical proteins | 13.16 |
| **Mobile and extrachromosomal element functions** | **0** | **Mobile and extrachromosomal element functions** | **0** |
| **rRNA and stable RNAs** | **0** | **rRNA and stable RNAs** | **0** |

- - 1. G.

| IA3902 (CAYNNNNNCTC) | | 11168 (ACNNNNNCTC) | |
| --- | --- | --- | --- |
| Role category | Percent methylated genes per category | Role category | Percent methylated genes per category |
| **Small molecule degradation** | **50.00** | **Chemotaxis and motility** | **40.48** |
| Transcription and RNA metabolism | 41.18 | Purines, pyrimidines, nucleosides, and nucleotides | 30.95 |
| Cellular processes | 27.59 | Transcription and RNA metabolism | 29.41 |
| General function | 21.47 | DNA metabolism, replication, and repair | 27.78 |
| Energy metabolism | 20.19 | Transport and binding proteins | 23.91 |
| DNA metabolism, replication, and repair | 20.00 | Cell envelope | 19.46 |
| Amino acid biosynthesis | 19.12 | Amino acid biosynthesis | 19.40 |
| Signal transduction | 18.75 | Protein synthesis | 19.38 |
| Biosynthesis of cofactors, prosthetic groups, and carriers | 18.18 | Energy metabolism | 19.05 |
| Conserved hypothetical proteins | 17.91 | Central intermediary metabolism | 18.75 |
| Protein fate | 17.39 | Pseudogenes/degenerate CDS | 17.65 |
| Fatty acid biosynthesis | 17.24 | Small molecule degradation | 16.67 |
| Central intermediary metabolism | 16.67 | Cellular processes | 16.36 |
| Transport and binding proteins | 16.55 | Conserved hypothetical proteins | 15.67 |
| Cell envelope | 14.88 | Protein fate | 15.56 |
| Purines, pyrimidines, nucleosides, and nucleotides | 14.29 | General function | 14.79 |
| Chemotaxis and motility | 11.90 | Hypothetical proteins | 14.04 |
| Regulatory functions | 11.54 | Regulatory functions | 12.00 |
| Hypothetical proteins | 11.20 | Fatty acid biosynthesis | 10.71 |
| Protein synthesis | 10.08 | Biosynthesis of cofactors, prosthetic groups, and carriers | 6.76 |
| Pseudogenes/degenerate CDS | 7.14 | Signal transduction | 6.25 |
| **Mobile and extrachromosomal element functions** | **0.00** | **Mobile and extrachromosomal element functions** | **0.00** |
| **rRNA and stable RNAs** | **0.00** | **rRNA and stable RNAs** | **0.00** |

- - 1. H.

| IA3902 (GAAGAA) | | 81-176 (GGRCA) | |
| --- | --- | --- | --- |
| Role category | Percent methylated genes per category | Role category | Percent methylated genes per category |
| **Pseudogenes/degenerate CDS** | **61.11** | **Central intermediary metabolism** | **75.00** |
| Signal transduction | 60.00 | Transcription and RNA metabolism | 64.71 |
| Small molecule degradation | 53.57 | Protein fate | 62.22 |
| Transport and binding proteins | 50.00 | DNA metabolism, replication, and repair | 61.54 |
| Regulatory functions | 50.00 | Small molecule degradation | 60.00 |
| DNA metabolism, replication, and repair | 47.62 | rRNA and stable RNAs | 58.82 |
| Fatty acid biosynthesis | 47.06 | Transport and binding proteins | 57.69 |
| Transcription and RNA metabolism | 45.76 | Purines, pyrimidines, nucleosides, and nucleotides | 57.50 |
| Central intermediary metabolism | 45.45 | General function | 56.79 |
| Protein synthesis | 44.83 | Energy metabolism | 56.31 |
| Amino acid biosynthesis | 43.27 | Chemotaxis and motility | 54.67 |
| Biosynthesis of cofactors, prosthetic groups, and carriers | 41.38 | Amino acid biosynthesis | 52.24 |
| Chemotaxis and motility | 41.18 | Biosynthesis of cofactors, prosthetic groups, and carriers | 51.95 |
| Cell envelope | 38.60 | Regulatory functions | 50.00 |
| Protein fate | 37.50 | Protein synthesis | 49.61 |
| Energy metabolism | 35.66 | Fatty acid biosynthesis | 48.28 |
| General function | 33.33 | Cell envelope | 43.98 |
| Purines, pyrimidines, nucleosides, and nucleotides | 28.57 | Cellular processes | 36.54 |
| Cellular processes | 26.40 | Hypothetical proteins | 33.71 |
| Hypothetical proteins | 23.88 | Conserved hypothetical proteins | 33.61 |
| Conserved hypothetical proteins | 20.69 | Signal transduction | 25.00 |
| **Mobile and extrachromosomal element functions** | **0.00** | **Mobile and extrachromosomal element functions** | **0.00** |
| **rRNA and stable RNAs** | **0.00** | **Pseudogenes/degenerate CDS** | **0.00** |
